# LGBTQ+ Adult Sexual Violence Critical Scoping Review: Insights into Victimization and Perpetration

**DOI:** 10.1177/15248380241311928

**Published:** 2025-01-22

**Authors:** Sophie Hindes, Jessica Ison, Bianca Fileborn

**Affiliations:** 1 La Trobe University, Bundoora, VIC, Australia; 2 The University of Melbourne, Parkville, VIC, Australia

**Keywords:** sexual violence, LGBTQ+, queer, transgender, victimization

## Abstract

The existing research on sexual violence has primarily concentrated on instances where cisgender, heterosexual men have perpetrated sexual violence against cisgender, heterosexual women, with knowledge about LGBTQ+ people underdeveloped. However, there is a growing body of literature examining the experiences of LGBTQ+ people. No previous review has critically synthesized both quantitative and qualitative scholarly studies on adult LGBTQ+ sexual violence globally. In this scoping review, we provide a comprehensive overview of current research by examining the prevalence of victimization and perpetration of adult LGBTQ+ sexual violence. To do this, an extensive search of the Discovery database was conducted, and studies published between 1990 and September 2021 were included. The final sample comprised 108 papers focused on LGBTQ+ participants’ experiences of adult sexual violence. Across studies, LGBTQ+ people were found to experience high rates of sexual violence, most commonly perpetrated by men, with rates the highest among transgender and gender-diverse people and bisexual women. However, the body of research is limited as it largely consists of quantitative studies from the United States with differing methodological approaches for measuring sexual violence and gender and sexuality, making it difficult to draw comparisons across studies. We propose recommendations to enhance future research on LGBTQ+ sexual violence.

## Introduction

Sexual violence has been the subject of growing concern and scrutiny over the past two decades, with research and theoretical frameworks in this field overwhelmingly focused on sexual violence perpetrated by cisgender, heterosexual men against cisgender, heterosexual women (Fileborn, 2012; Mortimer et al., 2019). Despite the increasing attention paid to sexual violence, research on LGBTQ+ people’s experiences remains underdeveloped. Mainstream sources of data on sexual violence, such as crime victimization surveys and police crime statistics, rarely collect information about sexuality or gender diversity (Messinger & Koon-Magnin, 2019). Nonetheless, there has been a consistent and growing stream of studies that have been published over the past 30 years, providing some insight into LGBTQ+ people’s experiences of sexual violence. Notably, this body of research suggests that LGBTQ+ people face disproportionately high levels of sexual violence (Felix et al., 2021; Fileborn, 2012). While this research demonstrates that there is certainly similarity with the experiences of cisgender, heterosexual women survivors, there are also aspects of LGBTQ+ people’s experiences that are distinct, with these differences often driven by heteronormativity and cisnormativity (Fileborn, 2012; Mortimer et al., 2019). For instance, stereotypes about bisexual people as hypersexual or sexually promiscuous shape their experiences of sexual violence as they may be perceived to be automatically consenting and are sexually objectified (Flanders et al., 2020). Similarly, the fetishization and marginalization of transgender women works to legitimize various forms of sexual harm against them, particularly if they are also racially festishized (Ussher et al., 2020).

In this article, we aim to draw together this growing body of work to critically reflect on the current state of knowledge on the prevalence of sexual violence victimization against LGBTQ+ adults, and the contexts and characteristics of perpetration against LGBTQ+ people. We use LGBTQ+ as an umbrella term to refer broadly to sexuality and gender-diverse people. However, when discussing individual studies, we draw on the terminology used by the study authors. This includes the labels individual studies used to describe sex, gender, and sexuality. We note that some of this terminology is now dated, and some terms may be considered harmful. For example, though a previously used term and a term used by some authors in this review, homosexual as a descriptor of a person is now considered to be stigmatizing and has caused harm (American Psychological Association, 2022). We have not included people with intersex variation as intersex communities have advocated for their need to be addressed independently from the LGBTQ+ umbrella (Carpenter, 2023). Intersex variation was also not a demographic characteristic examined by studies within our search terms. Additionally, while many studies report on “men”, “women,” “males,” and “females” without specifically asking about trans or gender-diverse status, unless a study explicitly states the inclusion of trans and gender-diverse individuals, we have not categorized them as such. While it is possible that trans individuals were included as participants, the available data do not confirm this, and trans people were not specifically addressed as a category of analysis, effectively rendering them invisible in the research. This limitation underscores the need for more precise reporting on the inclusion of diverse gender identities in future studies.

Several recent reviews have explored sexual violence victimization against various segments of the LGBTQ+ population or sexual violence in specific contexts. Rothman et al. (2011) conducted a systematic review analyzing victimization prevalence rates in United-States-based quantitative studies, focusing on gay, lesbian, and bisexual populations. Anderson et al. (2022) synthesized quantitative literature on sexual violence perpetration among sexual minority college men in the United States and Canada. However, across the 24 studies they analyzed, none reported on rates of perpetration by sexuality. Some reviews have examined LGBTQ+ sexual violence within specific contexts, including Klein et al.’s (2022) review of quantitative and qualitative research in college/university settings in the United States and Edwards et al.’s (2022) review of quantitative and qualitative English-language publications discussing LGBTQ+ sexual assault disclosure.

Additionally, no other review has drawn together the research on transgender and gender-diverse sexual victimization. We aimed to rectify these gaps through a scoping review of the global (published in English) academic peer-reviewed literature on adult LGBTQ+ sexual violence, including that which has been published on trans and gender-diverse populations. Due to the large number of studies, we address the findings over two papers. In this first paper, we report on the prevalence of sexual violence experienced by LGBTQ+ people, and the perpetration of sexual violence by and against LGBTQ+ people. In the second paper (Ison, Hindes & Fileborn, 2025), we analyze the risk factors for LGBTQ+ sexual violence victimization. Across both papers, we examine the current state of knowledge and critically examine key gaps and limitations in the literature. We also note that while this scoping review focuses on academic peer-reviewed literature, much of the pioneering research on LGBTQ+ sexual violence has been led by community-sector and nonprofit organizations, and while this is not included in this scoping review, these studies provide further evidence of the high rates of sexual violence experienced by LGBTQ+ people (see for instance, Hill et al., 2020; Layard et al, 2022).

## Measuring and Understanding Sexual Violence Victimization and Perpetration

Before presenting the findings of our review in relation to prevalence, it is helpful to first situate this work within broader debates regarding how sexual violence is defined and measured. The phenomenon of sexual violence is not a fixed or stable category (Fileborn, 2016; Herbele & Grace, 2009). Across studies, there can be different definitions or understandings operationalized in terms of “what counts” as sexual violence. For example, researchers may draw on survivor-centered or experiential understandings of sexual violence, Kelly’s (1988) influential continuum model of sexual violence, legal definitions of sexual violence (which can differ substantially across jurisdictions), and so forth (Westmarland & Bows, 2019). These conceptual and definitional slippages across research, while perhaps unavoidable, present significant challenges in drawing comparisons across studies—a challenge that emerged in the course of our own review, as we discuss later. These definitional and linguistic choices also have implications for which and whose experiences of sexual violence are captured within a study and, therefore, what is reflected within prevalence rates (Westmarland & Bows, 2019).

Intertwined with these definitional challenges is the epistemological question of measurement. That is, how do we come to “know” about, document, or measure experiences of sexual violence? Questions of measurement have been subject to much debate within sexual violence scholarship, and there is again no consensus across studies as to what approach is taken, though best practice approaches have been established (Westmarland & Bows, 2019). The lack of a standardized approach can make it difficult to draw conclusions about prevalence rates across sexual violence research more broadly. Again, *how* these questions are asked has significant implications in terms of whether a participant will disclose experiences of sexual violence, or recognize their experiences as “counting.” For example, some studies measure victimization and perpetration through single questions that focus on legal concepts rather than behavioral descriptions, for example, “In the last 12 months, have you been the victim of sexual assault?”. As numerous feminist researchers have argued, this style of questioning is likely to result in participant nondisclosure, as survivors do not always label their experiences as “sexual assault” or “rape” (Walby et al., 2017; Westmarland & Bows, 2019). This is particularly important to consider in the context of LGBTQ+ sexual violence. Myths and stereotypes about sexual violence as something that only occurs in the contexts of heterosexual relationships and to cisgender bodies can render LGBTQ+ people’s experiences invisible within dominant language and frameworks for understanding sexual violence (Mortimer et al., 2019).

Measurement tools such as the Sexual Experiences Survey (SES), developed by Koss et al. (2007), redress some of these issues, and the SES is now widely adopted in quantitative research on sexual violence. The SES assesses self-reported victimization and perpetration utilizing behaviorally specific descriptions of acts and tactics to measure victimization and perpetration across a continuum of sexual experiences from sexual coercion to rape (Koss et al., 2007). Originally designed to measure women’s victimization and men’s perpetration, it has since been changed to use gender-neutral language to rectify this heterosexist framing (Canan et al., 2020; Koss et al., 2007). While measurement tools such as the SES may provide more robust insights into participants’ experiences, as we outline further in our findings, such tools were not always used to measure sexual violence. Where the SES was utilized, it was not consistently adapted to be more inclusive of LGBTQ+ people (such as removing reference to specific body parts that reflect heteronormative understandings of sexual violence).

Compared to victimization studies, there is significantly less known about the perpetration of sexual violence against LGBTQ+ people, particularly whether LGBTQ+ people are perpetrated against by other LGBTQ+ people or heterosexual cisgender men (Anderson et al., 2021). Collecting data on perpetration against LGBTQ+ people is vital in order to understand the contexts in which LGBTQ+ people are experiencing violence and from whom. However, establishing robust datasets on perpetration is challenging, with primary insights in the literature derived from smaller-scale studies, particularly focusing on young people and college convenience samples (Anderson et al., 2021). We again see significant disparities in findings across studies due to variations in terminology, definitions, and measurement tools. For instance, Anderson et al. (2023) found that participants are more likely to admit to perpetration when the term “sexual assault” is used instead of “rape.” Acknowledgment also increases with a scaled response format compared to a yes/no format. Further, Anderson et al. (2021) discovered that using less explicit terms and behaviorally specific measures leads again to a notable rise in reported instances of sexual perpetration. Consequently, the framing of questions significantly influences disclosure, complicating the establishment of definitive conclusions on perpetration rates.

Collectively, our brief overview of the literature here suggests that any attempt to establish the prevalence of sexual violence victimization and perpetration is fraught, with the figures arrived at arguably an artifact of the study design and definitional scope. We do not say this to disparage attempts to quantify the phenomena of sexual violence, which remains an important endeavor in many respects (Westmarland & Bows, 2019). Rather, our discussion here has aimed to turn a critical lens on the process of knowledge production in sexual violence research, and to highlight the need for methodological choices that enable a more robust account of participants’ experiences, particularly (but by no means only) for LGBTQ+ victim-survivors. Many of the methodological concerns outlined here were apparent across the studies reviewed, and we turn now to outline our study methods and key findings.

## The Current Study

This paper is part of a larger scoping review on adult LGBTQ+ sexual violence. In this paper, we focus on studies examining the prevalence of victimization and perpetration. In the second accompanying paper, we look at the risk factors for LGBTQ+ people experiencing sexual violence (Ison, Hindes & Fileborn, 2025). We move on now to outline the methodological approach undertaken in conducting this review, before presenting key findings from across the literature. We close by offering critical reflections on the state of the field and consider future directions for research.

## Methods

### Search Strategy

In this study, we aimed to explore the current state of knowledge on LGBTQ+ people and adult sexual violence. A scoping review methodology was used to synthesize the growing field of research. Scoping reviews are a rigorous and transparent methodology for mapping areas of research (Pham et al., 2014). This study followed the extended guidelines for a scoping review outlined by Levac et al. (2010, p. 3), which built on Arksey and O’Malley’s (2005) six-stage framework. These steps include: identifying the research questions and relevant studies, charting the data, collating and synthesizing, and reporting the results. We used the Preferred Reporting Items for Systematic Reviews and Meta-Analyses (PRISMA) framework for scoping reviews to report our results (Tricco et al., 2018). Using the terms in Table 1, we searched for any studies that were published between 1990 and September 28, 2021 when the search was conducted.

**Table 1.** Search Terms.

Queer OR LBG* OR LGB* OR Lesbian OR bisexual OR trans OR transgender OR transwoman OR transman OR transmasc* OR transfem* OR non-binary OR “gender diverse” OR “sexual minority” OR pansexual OR homosexual OR gay OR “men who have sex with men” OR demisexual OR “gender fluid” OR transexual OR transsexual OR “beyond binary” OR “women who have sex with women”	AND	“sex* violence” OR rape OR “sex* assault” OR consent OR “sex* coercion” OR “unwanted sex” OR “sex crime” OR “sex* scripts” OR “date rape” OR “image-based abuse” OR “technology-facilitated violence” OR “sex* victimi?ation” OR “sex* exploitation” OR “unlawful sex” OR “woman-to-woman sexual violence” OR “men victims” OR “male victims” OR survivors OR “attempt* rape” OR “acquaintance rape” OR “force* sex” OR “non-consen* sex” OR “sex* offence” OR “sex* offense” OR “sex* attack” OR “unlawful sex* conduct” OR “sex* misconduct” OR “indecent* assault*” OR sextortion OR “image based abuse” OR sexting OR “non consent* sexting” OR “interpersonal trauma” OR “rape culture” OR “gender* violence” OR “gender based violence” OR “intimate partner sexual violence” OR “sex* abuse”

Studies were included if they met the following inclusion criteria, which were developed to narrow the scope of the review while capturing papers that had a strong focus on LGBTQ+ populations and sexual violence:

- 50% or more of the paper about sexual violence (to capture studies where sexual violence was the focus of analysis, not an “add on,” which is often the case in, for example, studies of domestic violence or HIV research)
- 50% or more of the paper relates to LGBTQ+ participants (not related to sample size but focus of analysis)
- Paper primarily focuses on adult (age 14+) experiences of victimization or perpetration
- English-only peer-reviewed articles

We excluded any studies that:

- Had less than 50% focus on sexual violence (for example, studies that were primarily about domestic violence but discussed sexual violence in passing)
- Had less than 50% focus on LGBTQ+ experiences (for example, studies that had some LGBTQ+ participants but LGBTQ+ sexual violence was not a primary focus of the article, or studies on same-sex sexual violence that was not LGBTQ+ specific, for example, heterosexual “male rape”)
- Were primarily concerned with child sexual abuse, or where it was unclear whether participants were reporting on sexual violence that had occurred in childhood or adulthood (e.g., some “lifetime prevalence” studies)
- Were theses or dissertations
- Were theoretical or related to perceptions, rather than experiences of sexual violence victimization and perpetration
- Were scoping and narrative reviews and non-peer-reviewed material such as conference papers, commentaries and editorials, books and websites
- Were focused exclusively on sexual harassment

### Study Selection and Data Extraction

**Figure 1.**
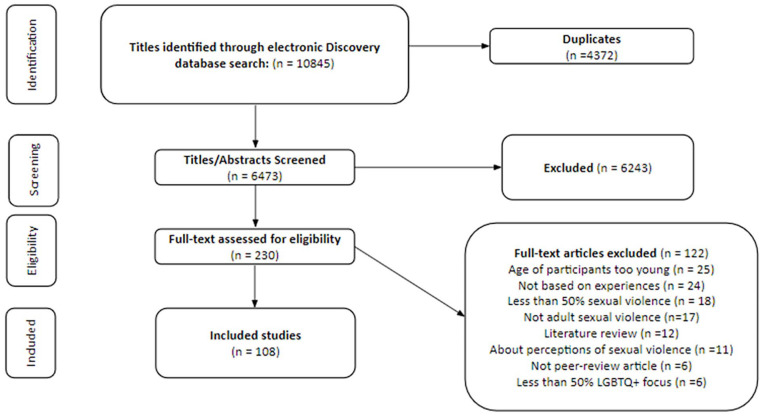
PRISMA flowchart. PRISMA: Preferred Reporting Items for Systematic Reviews and Meta-Analyses.

The search took place in September 2021 using the Discovery database. We used our key terms (Table 1) to search both the title and abstract of papers, returning a result of 10,845 papers. As reflected in the PRISMA flowchart in Figure 1, 10,845 papers were downloaded into Covidence (Veritas Health Innovation, 2021), 4,375 duplicates were removed, and 6,475 papers remained. Following our inclusion and exclusion criteria as outlined above, the first and second authors independently screened the titles and abstracts, rejecting 6,243 papers as irrelevant. A full-text review of 232 papers was performed by all 3 authors, where we applied the inclusion/exclusion criteria, and differences were resolved through discussion. The final selection of studies included 108 papers for final extraction (see Supplemental Appendix A for a list of included studies). All three authors extracted the final data, with the key elements of studies extracted using Covidence software. The results were then exported into a spreadsheet for analysis.

All three authors then analyzed the final sample of studies and individually identified the key themes emerging across the literature. The authors then met to discuss and decide on the key themes for analysis. This was not an attempt to quantify themes; rather, we took a critical approach to the scoping review, drawing out the recurring themes that warranted investigation. When mapping out the key themes, studies focused on victimization prevalence dominated the results and, to a lesser extent, studies on perpetration prevalence and contexts. All three authors also noted a focus across the literature relating to risk factors for experiencing sexual violence, which we explore in paper 2. After deciding on the key themes through discussion and reflection, the papers were then sorted into these thematic categories for analysis. Due to the number of papers and themes, we have reported the findings across two papers to ensure depth of analysis.

## Results

### Synthesis of Findings

This scoping review systematically synthesized the state of knowledge on adult LGBTQ+ sexual violence in the global peer-reviewed literature published in English. In Table 2, we provide a descriptive overview of the studies included in the final sample according to methods, study location and year of publication (see Supplemental Appendix B for a detailed summary of all research studies). These findings highlight that research on this topic has overwhelmingly been conducted in the United States utilizing quantitative methodologies. They also demonstrate the explosion of research that has occurred in this field, with 75 articles published from 2015 onwards, compared with the 33 publications in the decades spanning 1994 to 2014. Table 3 provides a general breakdown of participant groups across studies and illustrates that studies disproportionately focus on sexuality-diverse participants (typically gay, lesbian, and bisexual people). There was a relative paucity of work focusing on making gender-diverse and transgender people visible or on diverse sexualities such as pansexual or queer.

**Table 2.** Descriptive Summary of Papers.

Study Methods	Study Location		Year of Publication	
Quantitative *n* = 70	United States *n* = 72	New Zealand *n* = 3	2021 *n* = 17	2017 *n* = 10
Qualitative *n* = 24	Canada *n* = 8	Germany *n* = 3	2020 *n* = 24	2016 *n* = 6
Mixed *n* = 14	Australian *n* = 5	Other *n* = 17	2019 *n* = 6	2015 *n* = 6
			2018 *n* = 6	1994–2014 *n* = 33

**Table 3.** Participant Samples by Gender and Sexuality.

Participant Sample by Gender and Sexuality	Number of Papers
LGBQ+ men	22
LGBQ+ women	13
Transgender/NB people	12
LGBQ+ women; LGBQ+ men; heterosexual women; heterosexual men	11
LGBQ+ women; LGBQ+ men	10
LGBQ+ women; LGBQ+ men; transgender/NB people	10
LGBQ+ women; heterosexual women	10
LGBQ+ women; LGBQ+ men; heterosexual women; heterosexual men; transgender/NB people	9
LGBQ+ men; transgender/NB people	4
LGBQ+ women; transgender/NB people	3
LGBQ+ women; LGBQ+ men; heterosexual women	2
LGBQ+ women; heterosexual women; transgender/NB people	1
LGBQ+ men; heterosexual men; transgender/NB people	1

Table 4 shows the most common settings of studies. These include institutional settings such as universities or prisons. However, we note that there are an additional 28 studies that recruited university/college students as participants but did not focus on sexual violence occurring within a university/college context per se. This underscores the extensive utilization of college student samples throughout the research.

**Table 4.** Settings of Studies.

Setting	Studies
University/college campus setting	Coulter and Rankin (2020); Martin-Storey et al. (2018); Seabrook et al. (2018); Tilapaugh (2016)
Military	Beckman et al. (2018); Gurung et al. (2017); Lucas et al. (2018); Schuyler et al. (2020)
Prison	Jenness and Sexton (2021); Jenness et al. (2019); Ratkalkar and Atkin-Plunk (2020); Wilson et al. (2017)
Sex work	Peitzmeier et al. (2015); Seelman (2015); Semple et al. (2017); Shaw et al. (2012); Strike et al. (2001); Ussher et al. (2020)
Work other than sex work	Twinley (2017)
Licensed venues	Fileborn (2014)
Social media	Noack-Lundberg et al. (2020)
Intimate partner sexual assault	Gabbay and Lafontaine (2020); Heintz and Melendez (2006); Toro-Alfonso-Alfono and Rodriquez-Madera (2004); Wells et al. (2016)

Due to the large number of studies, we provide a snapshot of findings from selected papers and outline the general trends observed across the papers included in our review. We cover the following themes: prevalence of sexual violence victimization for LGBTQ+ people; sexual violence perpetration in LGBTQ+ contexts. We selected articles that illustrate the breadth of findings across each theme. This includes those reporting the highest and lowest prevalence rates, as well as examples representing mid-range rates. Additionally, we highlighted specific trends identified within gender or sexuality subgroups throughout the scoping review, drawing on a diverse range of papers to demonstrate these patterns across different study contexts.

### Prevalence of Sexual Violence Victimization for LGBTQ+ People

Studies attempting to document and quantify the prevalence of sexual violence victimization were some of the most common in our sample. We have divided our analysis into key groups that were the focus of analysis across papers. First, we look at studies where LGBTQ+ people are compared with their heterosexual counterparts. In general, these studies tended to collapse LGBTQ+ people together for comparison purposes, and many did not ask about participants’ gender beyond a binary woman/man framework, potentially excluding or obscuring transgender and gender-diverse people. Some studies did ask inclusive questions about gender identity but were unable to include transgender and gender-diverse participants in their analysis due to the small sample size (e.g., Potter et al., 2020). Second, we look at studies that focus exclusively on sexuality diverse participants, that is, studies focused only on sexuality rather than gender diversity. Third, we look at studies that included transgender and gender-diverse people. We acknowledge that this is, to some extent, a false distinction given that transgender and gender-diverse people may also be sexuality diverse. However, we have separated out findings relating to this cohort to ensure that the small body of work on transgender and gender-diverse people is foregrounded. It was also rare for studies to examine the intersection between gender and sexual diversity, and the few studies that did undertake a more intersectional analysis rarely reported significant differences in rates of sexual violence for transgender people of different sexualities (e.g., Coulter et al., 2017). More commonly, these studies either focused on trans and gender-diverse people as a standalone cohort or compared the experiences of transgender and cisgender participants (e.g., Johnson et al., 2016).

#### Victimization Prevalence in Studies Comparing LGBTQ+ and Heterosexual Participants

The studies included in our sample indicate LGBTQ+ prevalence rates of sexual violence victimization in adulthood are typically higher than those of cisgender, heterosexual samples where such comparison occurred (Coulter et al., 2017; Gilmore et al., 2021; Krahé and Berger, 2013; Palmer, 2021; Potter et al., 2020). For example, Gilmore et al.’s (2021, p. 6) quantitative study with 758 U.S. college students found that 47.8% of sexual minority participants had experienced some form of sexual violence since age 14, compared with 31.4% of heterosexual participants. In another example, Synder et al.’s (2018, p. 7) study of 43,000 college students in the United States found that gay (11%), bisexual (7%), and questioning (7%) males reported experiencing sexual assault at over twice the rate of heterosexual males (3%). For females, they found that while lesbians (8.20%) had a similar rate to heterosexual (8.32%) females, both bisexual (18.14%) and questioning (13.32%) females reported significantly higher rates of sexual assault.

Krahé and Berger’s (2013, p. 396) German study examined self-reported experiences of sexual victimization since age 14 in a sample of 2,149 first-year university students. Regardless of sexuality, women (35.9%) reported significantly higher rates of sexual victimization than men (19.4%) (see also Gilmore et al., 2021). An exception to this trend is noted in the study by Waldner-Haugrud and Vaden Gratch (1997), where women participants reported lower levels of sexual violence; however, this could be attributed to the study’s focus solely on unwanted sexual behavior by a lesbian or same-sex partner (see discussion on perpetration below). Krahé and Berger (2013) also found that participants who reported having both heterosexual and same-sex sexual encounters reported significantly higher victimization rates compared with exclusively heterosexual or same-sex attracted participants. Notably, women who had sex with men and women “had the highest victimization rate (39.7%), compared with women who only had sex with men (31.1%), followed by men who had sex with both men and women (25%) and men who only had sex with women (14.6%)” (Krahé & Berger, 2013, p. 400).

That bisexual women face the highest risk of sexual violence was, overall, a consistent finding across studies in the sample (e.g. Coulter et al., 2017). Likewise, studies typically found that lesbian and sexual minority women (including studies that analyzed bisexual and lesbian women as a single group) reported higher rates of sexual violence than heterosexual women (Satinsky & Jozkowski, 2014; Stoddard et al., 2009). However, there were some exceptions to this trend, for example, Long et al.’s (2007, p. 692) U.S.-based survey on male-perpetrated adult sexual assault found that heterosexual women (72.6%) experienced higher levels of “completed rape” than bisexual (67.8%) or lesbian (58.7%) women. The authors suggest that the lower prevalence rates of lesbian and bisexual women may be an artifact of excluding their experiences of sexual violence perpetrated by other women, and by focusing on the women’s “most serious” experience, rather than lifetime prevalence of sexual violence—highlighting how study design shapes victimization prevalence rates.

#### Victimization Prevalence of Sexuality Diverse Participants

We move on now to discuss findings from studies that focused exclusively on sexuality diverse participants. Most of these studies focused on lesbian, gay, and bisexual samples. One of the higher-end estimates of the lifetime prevalence of adult sexual assault for gay and bisexual men comes from Hequembourg et al.’s (2015) U.S.-based study with 183 gay and bisexual cisgender men aged 18 to 35 years old, recruited in Buffalo, New York State, as part of a larger study about risk and protective factors associated with substance use and physical and sexual victimization Approximately two-thirds of participants had at least one experience of sexual assault in adulthood, while 30.6% said they had experienced “sexual coercion, attempted rape, or rape in the past six months” (Hequembourg et al., 2015, p. 288).

Hickson and Davies’s (1994, p. 286) U.K.-based questionnaire with 930 gay men provided some early insights into participants’ first experiences of nonconsensual sex, with 27.6% indicating they had ever experienced nonconsensual sex. Forced anal penetration was the most commonly reported experience (45.2%). However, participants were asked, “how old were you when you were first sexually molested or raped, that is subjected to sex without your consent?” (p. 285), and this question design may have prompted participants to disclose more stereotypically “serious” forms of sexual violence. Further, as participants were asked to discuss their *first* experience of sexual violence, the authors note that the findings inevitably over-represent incidents that occurred in childhood and adolescence. When reporting on some of the outcomes, the study did not clearly distinguish between sexual violence experienced in childhood versus adulthood. While some forms of violence were reported as occurring across adolescence and adulthood (e.g., for participants “molested by their regular sexual partner,” the age at assault ranged from 15 to 37), in other cases, the ages ranged dramatically. For example, participants who reported incidents perpetrated by a colleague or acquaintance ranged from 7 to 49 years.

Other studies, such as Hequembourg et al.’s (2011, p. 9) U.S.-based study, found lower rates of sexual victimization among gay men or men who have sex with men, with 14.2% of the 634 men in this study indicating that they had experienced sexual violence as an adult. However, this study relied on a single question on sexual victimization (whether the participant “had ever been forced or frightened by someone into doing something sexually that you did not want to do”) (p. 6), with this measure likely to undercount the extent of sexual victimization. Waldner-Haugrud and Vaden Gratch’s (1997, p. 92) U.S.-based study with lesbian women and gay men found that over half of participants (52%) had experienced at least one incident of sexual coercion from a same-sex partner, with unwanted penetration the most commonly reported experience for both gay men (55%, *n* = 146) and lesbians (50%, *n* = 74).

However, the aforementioned finding that bisexual and lesbian women experience higher rates of sexual violence compared with heterosexual women and sexuality diverse men should be approached with some caution. It is currently unclear to what extent these findings represent a higher prevalence of sexual violence, or rather a higher rate of *acknowledging and labeling* experiences of sexual violence by LGBTQ+ women. Understandings of sexual violence are shaped by (hetero)gendered norms and rape myths, which often portray certain forms of sexual violence as an inevitable part of heterosexual interactions (Hindes & Fileborn, 2020). Rape myth acceptance has been shown to impact whether victim-survivors label their experiences as rape or sexual assault (Peterson & Muehlenhard, 2004). A study by Wilson and Newins (2019) of rape myth acceptance and rape acknowledgment found that individuals who identified as sexual minorities were significantly more likely to acknowledge or label their experiences as rape than those who identified as heterosexual. They found that sexual minorities were more likely to reject rape myths, which was associated with a higher likelihood of identifying themselves as rape victim-survivors. Another study by Schulze and Koon-Magnin (2017) similarly found that LGBQ people, particularly LGBQ women, exhibit lower rape myth acceptance.

Conversely, studies of nonconsensual and coercive sex among gay and bisexual men have found that there are stereotypes about consent, power, and masculine sexuality in sex between men that may normalize or minimize nonconsensual sex (Braun et al., 2009b; Ford & Becker, 2020; Hickson & Davies, 1994). For instance, Braun et al. (2009b) found that stereotypes about gay men as hypersexual, and gendered tropes depicting men as having a high sex drive, can mean that there is a lack of counter-discourse about sexual assault and coercion between men. In turn, these stereotypes and discursive constructions can create barriers for gay men in recognizing or reporting their experiences. As such, it is difficult to disentangle the extent to which LGBTQ+ people may more readily recognize and label experiences of sexual violence, and the extent to which this might differ within and across these diverse communities. From the studies included in the review, there is evidence of *both* greater acknowledgment of sexual violence *and* the normalization and minimization of sexual violence for some LGBTQ+ people. Given this, the processes of acknowledging and labeling sexual violence within LGBTQ+ communities requires further exploration.

#### Victimization Prevalence of Transgender and Gender-Diverse Participants

Studies including transgender and gender-diverse participants typically drew on small convenience samples from the United States. Again, findings across studies suggest that transgender and gender-diverse people are subject to high rates of sexual violence, and higher rates of sexual violence compared with their cisgender counterparts (e.g., Griner et al., 2020; Paquette, 2019; though there were some exceptions to this, e.g., Peitzmeier et al., 2015). Coulter et al.’s (2017, p. 728) study with U.S. undergraduate students (*n* = 71,421) found that transgender participants reported the highest rate of sexual assault (20.9%) compared with participants of other genders.

Six studies illustrated the importance of undertaking an intersectional analysis when examining gender-diverse people’s experiences of sexual violence (Cook-Daniel & munson, 2010; Noack-Lunberg et al., 2020; Seelman, 2015; Staples & Fuller, 2021; Ussher et al., 2020). For example, Staples and Fuller’s (2021, p. 700) study with 235 transgender and nonbinary people in the United States considered the intersection of sexual assault severity, transgender visibility and race, finding “a significant interaction between race/ethnicity and being perceived as transgender.” Specifically, the severity of adult sexual assault increased only for transgender “participants of color” (defined as any race/ethnicity other than white) who were perceived as “visibly transgender,” as opposed to their white transgender counterparts. Cook-Daniels and munson’s (2010, p. 145) U.S.-based surveys with older transgender adults (age 50+, *n* = 53) found that 64% had experienced unwanted sexual touching. This study represents the only study to consider the experiences of older LGBTQ+ people, though it is worth noting that elder sexual abuse remains underexamined in general (Bows, 2018).

#### Methodological Limitations of Victimization Prevalence Studies

There are some significant limitations associated with how studies across our sample measured prevalence, gender, and sexuality, which make comparisons across studies difficult. Many studies drew on survey measures that were originally designed in relation to cisgender, heterosexual women survivors, and there is a lack of appropriate measurement tools that consider the sexual practices and contexts of LGBTQ+ people (Anderson et al., 2022; McKay et al., 2019). For instance, some used the SES unmodified to include LGBTQ+ participants, employing the same questions used in heterosexual populations. Others modified the SES to be more inclusive of LGBTQ+ genders and sexualities. However, these adaptations were often implemented differently by various research teams. For instance, while Flanders et al. (2020) removed references to specific genitals to enhance inclusivity for sexual minorities engaging with individuals of different genders (i.e., bisexuals), Heidt et al. (2005) took a different approach by adding references to specific genitals (e.g., anal or vaginal penetration, instead of the more generic term “intercourse”) to eliminate the association with vaginal–penile penetration to foster greater inclusivity. Although both approaches have validity in terms of inclusion (depending on the framing), not having a unified method for adapting the SES for LGBTQ+ participants means that there are different measures used across studies, making it difficult to draw comparisons between studies.

Other studies used single questions like “In the past year, how often were you (a) raped, (b) sexually molested, and (c) the victim of sexual harassment?” (Semple et al., 2017, p. 1013). These types of questions are not considered best practice as respondents may not recognize their experiences within these terms, and these terms are commonly understood in heteronormative ways (Fileborn, 2015; Mortimer et al, 2019). Rather, detailed behavioral descriptions, such as those used in the SES, are better able to capture experiences of sexual violence across the spectrum (Gavey, 2019; Walby et al., 2017).

Studies also varied in the timeframes and definitions of sexual violence that occurred in “adulthood.” For instance, some studies enquired about experiences across adulthood (e.g., Gilmore et al., 2021; Krahé and Berger, 2013), while others enquired about experiences in the last 12 months (e.g., Coulter et al., 2017). “Adulthood” was variably considered to commence at age 14 or 18, while one study collapsed experiences across childhood and adulthood (Hickson & Davies, 1994). It is worth noting that many studies explored sexual violence “across the life course,” and a considerable number of studies had to be excluded due to the inability to differentiate between experiences that occurred in childhood or adulthood. This raises concerns as it hampers our ability to understand the distinct impacts and trajectories of sexual violence at different life stages. Additionally, this lack of clarity may obscure critical factors influencing victimization and perpetration dynamics in childhood sexual abuse versus adult sexual violence.

Our review also identified inconsistencies in the measurement and reporting of gender and sexuality, with studies often erasing or obscuring particular sexualities and gender diversity. For instance, the tools for measuring sexuality varied widely, often leading to oversimplifications that fail to capture the full spectrum of identities. For example, VanderLaan and Vasey (2009) used a Kinsey scale, classifying anyone with same-sex experiences or feelings as nonheterosexual, regardless of their self-identified sexual orientation. In contrast, Hughes et al. (2001) categorized women as lesbian if they identified as “mostly” or “only homosexual, lesbian, or gay.” Other studies offered more diverse identity labels, for example, Backhaus et al. (2021), provided a response scale with options such as Heterosexual, Lesbian, Gay, Bisexual, Queer, and Questioning but then collapsed these into a binary categorization of heterosexual or LGBQ for data analysis. These disparate approaches not only complicate comparisons across studies but also risk marginalizing or misrepresenting the experiences of those who do not fit neatly into the assigned categories.

Indeed, this practice of collapsing sexual and/or gender identities was common across studies. In some cases, studies collapsed findings across diverse groups, such as combining all gender and sexual minorities into a single category of analysis. While some studies did this to compare sexual and gender minorities against heterosexual populations, such as the Backhaus et al. (2021) study, for others this collapsing was often a result of small sample sizes that make robust statistical analysis difficult. However, this practice nonetheless effectively “washes out” the distinct experiences of certain LGBTQ+ communities in research (Klein et al., 2022; Rothman et al., 2011). For example, Tilley et al.’s (2020, p. 65) study grouped “all responses other than heterosexual” into the LGBT+ category to compare against heterosexual responses. However, as this categorization was solely based on sexuality and not gender, it remains unclear how they accounted for trans and gender-diverse participants, despite including the “T” in their acronym. Furthermore, the study analyzed how LGBT+ “peer norms” correlate with sexual victimization, finding that LGBTQ+ individuals “are more likely to have friends who approve of risky sexual behavior, which is correlated with sexual victimization” (p. 68). Drawing together all sexual minorities in this way assumes the existence of a unified and homogenous LGBTQ+ community with shared norms and experiences, disregarding the diverse ways LGBTQ+ individuals engage in sex and relationships (Ison, 2019). When juxtaposed with these “risk factors,” it also fosters stigmatizing ideas about LGBTQ+ communities, as expounded further in our second paper (Ison, Hindes & Fileborn, 2025). This is just one example of many that demonstrate the problems with collapsing LGBTQ+ people’s experiences across the literature.

At other times, studies collapsed some identities together, such as bisexual and lesbian women into “sexual minority women” (e.g., Hughes et al., 2010; Lopez & Yeater et al., 2021; Satinsky & Jozowski, 2014). This limits the ability to understand the unique experiences of these groups, including whether they encounter different contexts of victimization, such as their relationship to the perpetrator. Researchers need to consider what important differences are being lost or obscured and what assumptions are being made about LGBTQ+ people when they collapse distinct genders and sexualities together. Further, studies rarely documented whether the participant was “out” at the time they experienced sexual violence or who the perpetrator was, so it is difficult to know if the recorded experiences happened while a person was identifying as LGBTQ+ or within the context of an LGBTQ+ relationship. Therefore, we have limited understanding of how or whether the person’s LGBTQ+ identity was a factor in their experiences of sexual violence.

Collectively, despite methodological differences and limitations, it is clear across these studies that LGBTQ+ people experience high rates of adult sexual violence. Transgender, gender diverse, and bisexual women appear to experience disproportionately higher rates of sexual violence (with some variation across studies) than their other LGBTQ+ counterparts, and higher rates in comparison to cisgender, heterosexual men and women. In the next section, we report on the data in relation to sexual violence perpetration in LGBTQ+ contexts.

### Sexual Violence Perpetration in LGBTQ+ Contexts

We found that studies looking at the perpetration of sexual violence in LGBTQ+ contexts also faced significant methodological limitations. First, we found very limited literature on self-reported perpetration against LGBTQ+ individuals. A notable gap is that studies on perpetration often did not specify the identity of the victim-survivor or the context in which violence occurred, which hinders our understanding of perpetration against LGBTQ+ individuals. Although we identified eight papers that include self-reported experiences of perpetration by LGBTQ+ people (Fontanesi et al., 2020; Gabbay & LaFontaine, 2020; Ho et al., 2021; Krahé and Berger, 2013; Krahé et al., 2000, 2001; Strike, 2001; Vanderlaan & Vassey, 2009), the gender and/or sexuality of the victim-survivors and the relationship context were not reported in these studies. Consequently, it is unclear whether sexual violence was occurring between LGBTQ+ people or within LGBTQ+ relationships.

For example, Vanderlaan and Vassey (2009) studied 414 college students in Canada, including 112 nonheterosexual men and 89 nonheterosexual women. They found that 41.1% of the nonheterosexual men and 38.2% of the nonheterosexual women had perpetrated at least one physical act of sexual coercion against someone of the same sex, but the study did not report the victim-survivor’s sexuality. Similarly, Krahé and Berger’s (2013) study of 2,149 college students in Germany found that 5.1% of men with same-sex only contact and 22.1% of men and 7.6% of women with both same-sex and heterosexual contact had coerced someone into sex using verbal pressure. However, again, the sexuality of the victim-survivor was not identified. While a third paper did not report on perpetrator gender it found that 17% of the 455 LGBTQ+ adults they sampled reported perpetrating sexual violence in the past year, and 27% since the age of 14. However, the study did not clarify whether the victim-survivors in these contexts were LGBTQ+ (Ho et al., 2021, p. 1469).

Two other self-reporting papers (Krahé et al., 2000, 2001) derived from the same study that focused on homosexual men (*n* = 310) in Germany found that 20% of participants reported sexually aggressive behavior toward other men that met the legal definition of rape or sexual coercion. In these papers, the sexuality of the victim-survivor is not reported, but as the men surveyed are homosexual, it is presumed that this sexual violence is occurring “among homosexual men” (Krahe et al., 2001, p. 1402). These two papers also found a significant overlap between victimization and perpetration, with one-third of the homosexual men who perpetrated sexual aggression also having experienced sexual victimization. Strike et al.’s (2001) Canadian study also found a significant overlap between victimization and perpetration in their qualitative study of street-involved young adults (*n* = 50). However, they found this to be the case regardless of gender or sexuality. This overlap of victimization and perpetration highlights an area warranting further investigation.

Overall, across self-report studies, it was unclear whether LGBTQ+ perpetrators were enacting sexual violence against other LGBTQ+ people. However, some papers *assumed* that the victim-survivor was LGBTQ+ where self-reported perpetration involved same-gender contact. This lack of targeted research highlights a critical gap in the literature, emphasizing the need for future self-report studies to include detailed demographic information about both perpetrators and victim-survivors (where possible) and the context in which the sexual violence occurred.

Although most studies in our sample on victimization did not inquire about the perpetrators, those that did collect data from LGBTQ+ victim-survivors offered a deeper understanding of perpetration. These studies often included information on the perpetrators’ gender and the nature of their relationship with the victim-survivor, providing valuable insights into the dynamics of sexual violence against LGBTQ+ individuals. Across these studies, men were overall significantly more likely to be identified as the perpetrators. For example, Sigurvinsdottir et al.’s (2015, p. 641) study of heterosexual, lesbian, and bisexual women in the United States (*n* = 1,863) found that all groups were more likely to be perpetrated against by men than women, with men the perpetrators 96.5% of the time for heterosexual women, 92.6% of the time for bisexual women, and 89.4% of the time for lesbian women. Murchison et al.’s (2017, p. 230) study with LGBQ undergraduate students in the United States (*n* = 763) found that 87% of the 264 participants with unwanted sexual experiences reported male perpetrators. Cook-Daniel’s and munson’s (2010, p. 146) study with older transgender people in the United States (*n* = 53) found that 81% reported their perpetrators were male, 16% were female, and 2% were transgender. With the exception of Cook-Daniels and munson (2010) and Bedera and Nordmeyer (2020), all of the studies only reported whether perpetrators were male/female or men/women, either obscuring or excluding gender-diverse people as potential perpetrators. Perpetrators’ sexuality was also usually not reported. However, Bedera and Nordmeyer’s (2020) qualitative study with LGBTQ+ women in the United States (*n* = 40) found that out of the 67 sexual assault events captured in the data, eight were perpetrated by someone who at the time or has since come to identify as transgender or genderqueer and two were perpetrated by a pair (i.e., two people acting together) with differing gender expressions, with the remaining incidents perpetrated by cisgender men.

Overwhelmingly, victimization studies found that perpetrators were more likely to be someone the victim-survivor knew, such as a date, acquaintance, intimate partner, or family member. However, there were also some differences by victim-survivor gender and sexuality. For example, two studies found that lesbian women were more likely to be sexually assaulted by a family member as compared with other participants (Hughes et al., 2001; Sigurvinsdottir & Ullman, 2016b). Watson’s (2021, p. 238) mixed methods study with bisexual women in the United States (*n* = 532) found that 34.7% of participants whose perpetrators knew of their bisexual identity thought that it influenced the sexual assault, suggesting that biphobia may be a driving factor for perpetrators of sexual assault against bisexual women.

Overall, these studies consistently indicate that men make up the majority of perpetrators, regardless of the victim-survivor’s gender or sexuality. Perpetrators are also more likely to be someone the victim-survivor knows, such as an intimate partner, family member, or acquaintance. This is consistent with patterns found in sexual violence research against cisgender heterosexual women. However, more research is needed to understand the motivations and context of sexual violence perpetration against LGBTQ+ individuals, as while one study suggests biphobia may be a factor, we still have a limited understanding of how systems of oppression such as (e.g., racism, homophobia) shape perpetrators’ actions. There is also a limited understanding of sexual violence in LGBTQ+ relationships. So, while rates of sexual violence are high among LGBTQ+ populations, it is unclear from current studies which have analyzed perpetration how much of this violence is occurring *within* the context of LGBTQ+ relationships, from a perpetrator who is also LGBTQ+, and how/whether a victim-survivor’s LGBTQ+ identity shaped the perpetrator’s actions.

## Discussion

This scoping review examined 108 empirical studies on adult LGBTQ+ people’s experiences of sexual violence. In this paper, we reported on prevalence and perpetration (see, Ison, Hindes & Fileborn, 2025, for results on sexual violence risk factors). Our review highlights that research in this field is rapidly growing, and this body of work provides vitally important insights into the experiences of communities who have often been excluded from sexual violence research. However, we have identified limitations, particularly in relation to methodology, in the research to date. Collectively, these issues delimit the extent to which we can draw together findings and implications across studies, and the extent to which findings are likely to apply to LGBTQ+ communities outside of the United States, and particularly beyond young, predominantly white college student groups. While our scoping review took a global focus (limited to studies published in English), it is important to note that the significant majority of studies on LGBTQ+ adult sexual violence are from the United States. Consequently, the transferability of these findings to other global contexts, especially in lower or middle-income countries, and to older or non-college/university students remains uncertain.

Victimization prevalence rates varied significantly across studies. Such variation may be largely due to the differing measures used to assess victimization and sexuality, which complicates cross-study comparisons. Our scoping review also revealed a disproportionate focus on the experiences of gay, lesbian, and bisexual groups (albeit, often collapsed together), while transgender, gender-diverse, and other sexual minority participants were less frequently studied (at least explicitly). Some studies also combined the diverse experiences of different trans and gender-diverse identities into a generalized category of “transgender participants” (e.g., Coulter et al., 2017). While this approach effectively highlighted higher rates of sexual violence for trans and gender-diverse individuals compared to cisgender people, it obscured the unique experiences and contexts within this group. For example, Ussher et al. (2020) and Cook-Daniels & munson (2015) emphasized the distinctive experiences and contexts of sexual violence for transgender women, who often face fetishization, objectification, and heightened aggression. These studies also underscored the compounding effects of multiple marginalizations, with transgender women of color experiencing pervasive violence. To deepen our comprehension of the experiences of trans and gender-diverse individuals, there is a critical need for more research exploring nuanced differences from cisgender individuals and within trans communities. Furthermore, research should expand beyond the LGB (lesbian, gay and bisexual) categories to encompass sexual minority identities such as asexual, queer, pansexual, and others. It is crucial for research to consider intersectional dimensions that shape LGBTQ+ people’s experiences of sexual violence beyond just gender or sexuality.

Understanding perpetration against LGBTQ+ communities is also challenging. We found no studies on self-reported perpetration specifically against LGBTQ+ individuals, and in self-report studies with LGBTQ+ participants, it was unclear whether the victim-survivor was also LGBTQ+. While some victimization studies provided insights by asking about perpetrators, most did not, making it difficult to determine who is perpetrating against LGBTQ+ people. Studies on perpetration tend to suggest that men disproportionately perpetrate against LGBTQ+ communities. As Anderson et al. (2021) note, some research on intimate partner violence (e.g., Messinger et al., 2021; Miltz et al., 2019) found that rates of perpetration are lower than rates of victimization within LGBTQ+ communities. This raises the need to further disentangle the extent to which sexual violence is occurring *within* LGBTQ+ communities versus that perpetrated by cisgender, heterosexual men (Ison, 2019). This distinction is essential for developing targeted interventions. Failing to make these distinctions could lead to misconceptions that LGBTQ+ relationships or individuals are inherently more prone to sexual violence, despite high prevalence rates in LGBTQ+ populations.

Collectively, we found that there is a need for further research on LGBTQ+ experiences outside of the Global North (and particularly the United States), that is inclusive of the full spectrum of LGBTQ+ identities, and that includes qualitative methodologies that provide rich insights into the lived experiences of LGBTQ+ survivors. Qualitative methodologies may be particularly important for research with “hard to reach” or highly marginalized communities, given the challenges associated with recruiting “sufficient” participant numbers for statistical analysis. While prevalence rates offer insight into the extent of the issue, qualitative studies enable a deeper understanding of the social and cultural contexts surrounding sexual violence (Gavey, 2019). Again, this is particularly important for understanding the unique contexts of LGBTQ+ sexual violence, which may not fit into dominant heteronormative and cisnormative understandings (Mortimer et al., 2019). Additionally, how people describe their experiences qualitatively can be significantly different from how they label their experiences in survey data, with qualitative research providing important insights into how people describe and label their experiences (Testa et al., 2011).

Several areas are notably lacking in quantitative research and would benefit from further nuance in qualitative studies, this includes understanding sexual violence perpetration against LGBTQ+ people, research involving cohorts from low- and middle-income countries, studies focused on trans and gender-diverse populations, and investigations into how LGBTQ+ individuals recognize and label their experiences of sexual violence and the cultural norms around sexual consent in LGBTQ+ relationships and spaces (see, for example, Hindes, 2023; Webber, et al., 2024) which is crucial for understanding the high prevalence rates. Indeed, the small numbers of qualitative studies addressed in this paper add rich complexity to our understanding of sexual violence victimization (e.g., Bedera and Nordmeyer, 2020; Ussher et al., 2020), with more research such as this vital. Our next paper provides further insights into the field of LGBTQ+ sexual violence research, examining the sexual violence risk factors for LGBTQ+ people in our scoping review (Ison, Hindes & Fileborn, 2025).

## Conclusion and Limitations

This paper consolidates peer-reviewed research on the prevalence of LGBTQ+ adult sexual violence victimization and perpetration. It offers a comprehensive overview that extends beyond the conventional emphasis on sexual minorities, explicitly encompassing the experiences of trans and gender-diverse individuals. By broadening the scope beyond the often-exclusive focus on “LGB,” this paper aims to enhance our overall understanding of LGBTQ+ adult sexual violence. Our scoping review does, however, have some limitations. We did not include literature from the community-sector and nonprofit organizations as our aim was to synthesize academic literature. While we took a global focus, we were limited to English language papers due to the authors’ language limitations. Considering the different terminology across various fields, some papers may have been excluded from our study; however, we have undertaken a broad search with comprehensive search terms to minimize this possibility. Overall, this paper contributes a critical synthesis of LGBTQ+ sexual violence research and points to the key gaps and limitations of this body of research, particularly in terms of methodological limitations, providing scope for improving and extending future research on LGBTQ+ sexual violence.

## Key Findings

- Most research on LGBTQ+ sexual violence consists of quantitative studies from the United States with a majority utilizing white, college/university student participant groups.
- LGBTQ+ people experience high rates of adult sexual violence, with victimization rates the highest among bisexual women and transgender and gender-diverse participants. However, it is unclear whether these findings reflect higher rates of sexual violence, or higher rates of sexual violence acknowledgment.
- Sexual violence against LGBTQ+ people is most commonly perpetrated by men; however, it is unclear how much of this violence occurs within the context of LGBTQ+ relationships or from a perpetrator who is also LGBTQ+ as studies rarely enquire about the sexuality of the perpetrator, their relationship to the victim-survivor, and the context in which the violence occurred.
- Current research draws on vastly different definitions of sexual violence and different methodological approaches to measuring sexual violence, gender, and sexuality. This makes it challenging to draw comparisons across studies.

### Implications for Research, Policy and Practice

- Global expansion of research: The majority of LGBTQ+ sexual violence research is quantitative and originates from the United States. Future research should extend beyond this context to enhance understandings of LGBTQ+ sexual violence, particularly in low- and middle-income countries.
- Clarity on LGBTQ+ sexual violence needed: While LGBTQ+ individuals experience elevated rates of adult sexual violence, the nature of these findings remains ambiguous. It is unclear whether there are higher rates of violence as compared to heterosexual cisgender women, or increased rates of acknowledgment among LGBTQ+ people. Additionally, the lack of research on perpetration relationships and contexts and victim-survivors sexuality at the time of sexual violence obscures our ability to understand whether sexual violence is occurring within or outside of LGBTQ+ relationships. Future research must explore the nuanced aspects of LGBTQ+ people’s experiences and prioritize understanding perpetration.
- Intersectional approach needed: Current research often neglects intersecting forms of inequality and lacks inclusivity, particularly regarding trans and gender-diverse individuals and other sexualities beyond LGB people. Future research should adopt an intersectional approach to address these gaps comprehensively.
- Standardization of definitions, measurements, and methodologies: Current research utilizes varied definitions of sexual violence, diverse methodological approaches, and collapses LGBTQ+ identities in limiting and inconsistent ways, posing a challenge for cross-study comparisons. There is an urgent need for more consistent and inclusive measurements, and attention to the distinct identities within the LGBTQ+ spectrum to avoid collapsing diverse LGBTQ+ identities and experiences of sexual violence.

## Supplemental Material

sj-docx-1-tva-10.1177_15248380241311928 – Supplemental material for LGBTQ+ Adult Sexual Violence Critical Scoping Review: Insights into Victimization and Perpetration Supplemental material, sj-docx-1-tva-10.1177_15248380241311928 for LGBTQ+ Adult Sexual Violence Critical Scoping Review: Insights into Victimization and Perpetration by Sophie Hindes, Jessica Ison and Bianca Fileborn in Trauma, Violence, & Abuse

sj-docx-2-tva-10.1177_15248380241311928 – Supplemental material for LGBTQ+ Adult Sexual Violence Critical Scoping Review: Insights into Victimization and Perpetration Supplemental material, sj-docx-2-tva-10.1177_15248380241311928 for LGBTQ+ Adult Sexual Violence Critical Scoping Review: Insights into Victimization and Perpetration by Sophie Hindes, Jessica Ison and Bianca Fileborn in Trauma, Violence, & Abuse
