# Supplementary material for: LGBTQ+ Adult Sexual Violence Critical Scoping Review: Insights into Victimization and Perpetration
Source: Trauma Violence Abuse. 2025 Jan 22;27(2):305–18. doi: 10.1177/15248380241311928 (PMC12953667; doi:10.1177/15248380241311928)
Supplement: sj-docx-1-tva-10.1177_15248380241311928 – Supplemental material for LGBTQ+ Adult Sexual Violence Critical Scoping Review: Insights into Victimization and Perpetration [file sj-docx-1-tva-10.1177_15248380241311928.docx]

**Appendix A - Scoping Review Studies Reference List**

*Note: Those marked with an asterix (*) are referenced in the body of the article.*

Aspin, C., Reynolds, P., Lehavot, K., & Taiapa, J. (2009). An investigation of the phenomenon of non‐consensual sex among Maori men who have sex with men. *Culture, Health & Sexuality*, *11*(1), 35–49. https://doi.org/10.1080/13691050802483711

Backhaus, I., Lipson, S. K., Fisher, L. B., Kawachi, I., & Pedrelli, P. (2021). Sexual assault, sense of belonging, depression and suicidality among LGBQ and heterosexual college students. *Journal of American College Health*, *69*(4), 404–412. https://doi.org/10.1080/07448481.2019.1679155

Balsam, K. F., Lehavot, K., & Beadnell, B. (2011). Sexual Revictimization and Mental Health: A Comparison of Lesbians, Gay Men, and Heterosexual Women. *Journal of Interpersonal Violence*, *26*(9), 1798–1814. https://doi.org/10.1177/0886260510372946

*Beckman, K., Shipherd, J., Simpson, T., & Lehavot, K. (2018). Military Sexual Assault in Transgender Veterans: Results From a Nationwide Survey. *Journal of Traumatic Stress*, *31*(2), 181–190. https://doi.org/10.1002/jts.22280

*Bedera, N., & Nordmeyer, K. (2020). An Inherently Masculine Practice: Understanding the Sexual Victimization of Queer Women. *Journal of Interpersonal Violence*, *36*(23–24), 1. https://doi.org/10.1177/088626051989843

*Braun, V., Schmidt, J., Gavey, N., & Fenaughty, J. (2009a). Sexual Coercion Among Gay and Bisexual Men in Aotearoa/New Zealand. *Journal of Homosexuality*, *56*(3), 336–360. https://doi.org/10.1080/00918360902728764

Braun, V., Terry, G., Gavey, N., & Fenaughty, J. (2009b). “Risk” and sexual coercion among gay and bisexual men in Aotearoa/New Zealand-key informant accounts. *Culture, Health & Sexuality*, *11*(2), 111–124. https://doi.org/10.1080/13691050802398208

Connolly, D., Aldridge, A., Davies, E., Maier, L. J., Ferris, J., Gilchrist, G., & Winstock, A. (2021). Comparing Transgender and Cisgender Experiences of Being Taken Advantage of Sexually While Under the Influence of Alcohol and/or Other Drugs. *Journal of Sex Research*, *58*(9), 1112–1117. https://doi.org/10.1080/00224499.2021.1912692

*Cook-Daniels, L., & munson, michael. (2010). Sexual Violence, Elder Abuse, and Sexuality of Transgender Adults, Age 50+: Results of Three Surveys. *Journal of GLBT Family Studies*, *6*(2), 142–177. https://doi.org/10.1080/15504281003705238

*Coulter, R. W. S., Mair, C., Miller, E., Blosnich, J. R., Matthews, D. D., & McCauley, H. L. (2017). Prevalence of Past-Year Sexual Assault Victimization Among Undergraduate Students: Exploring Differences by and Intersections of Gender Identity, Sexual Identity, and Race/Ethnicity. *Prevention Science*, *18*(6), 726–736. https://doi.org/10.1007/s11121-017-0762-8

* Coulter, R. W. S., & Rankin, S. R. (2020). College Sexual Assault and Campus Climate for Sexual- and Gender-Minority Undergraduate Students. *Journal of Interpersonal Violence*, *35*(5–6), 1351–1366. https://doi.org/10.1177/0886260517696870

Crump, L., & Byers, E. S. (2017). Sexual well-being of sexual minority women in dating relationships who have experienced childhood sexual abuse and/or adolescent and adult sexual victimization. *Canadian Journal of Human Sexuality*, *26*(2), 163–173. https://doi.org/10.3138/cjhs.262-a4

DeLaney, E. N., Williams, C. D., Mosley, D. V, Hawn, S. E., & Dick, D. M. (2020). The Associations Between Sexual Victimization and Health Outcomes Among LGBQA College Students: Examining the Moderating Role of Social Support. *Journal of Interpersonal Violence*, *37*(11). https://doi.org/10.1177/0886260520978179

Drückler, S., Speulman, J., van Rooijen, M., & De Vries, H. J. C. (2021). Sexual consent and chemsex: a quantitative study on sexualised drug use and non-consensual sex among men who have sex with men in Amsterdam, the Netherlands. *Sexually Transmitted Infections*, *97*(4), 268–275. https://doi.org/10.1136/sextrans-2020-054840

Eisenberg, M. E., Lust, K., Mathiason, M. A., & Porta, C. M. (2021). Sexual Assault, Sexual Orientation, and Reporting Among College Students. *Journal of Interpersonal Violence*, *36*(1/2), 62–82. https://doi.org/10.1177/0886260517726414

Fernández-Rouco, N., Fernández-Fuertes, A. A., Carcedo, R. J., Lázaro-Visa, S., & Gómez-Pérez, E. (2017). Sexual Violence History and Welfare in Transgender People. *Journal of Interpersonal Violence*, *32*(19), 2885–2907. https://doi.org/10.1177/0886260516657911

*Fileborn, B. (2014). Accounting for Space, Place and Identity: GLBTIQ Young Adults’ Experiences and Understandings of Unwanted Sexual Attention in Clubs and Pubs. *Critical Criminology*, *22*(1), 81–97. https://doi.org/10.1007/s10612-013-9221-4

*Flanders, C. E., Anderson, R. E., & Tarasoff, L. A. (2020). Young Bisexual People’s Experiences of Sexual Violence: A Mixed-Methods Study. *Journal of Bisexuality*, *20*(2), 202–232. https://doi.org/10.1080/15299716.2020.1791300

Flanders, C. E., Anderson, R. E., Tarasoff, L. A., & Robinson, M. (2019). Bisexual Stigma, Sexual Violence, and Sexual Health Among Bisexual and Other Plurisexual Women: A Cross-Sectional Survey Study. *Journal of Sex Research*, *56*(9), 1115–1127. https://doi.org/10.1080/00224499.2018.1563042

*Fontanesi, L., D’Urso, G., Panzeri, M., & Pace, U. (2020). The Role of Attachment Style in Predicting Emotional Abuse and Sexual Coercion in Gay and Lesbian People: An Explorative Study. *Sexuality & Culture*, *24*(3), 504–515. https://doi.org/10.1007/s12119-019-09643-1

Ford, J. V. (2021). Unwanted Sex on Campus: The Overlooked Role of Interactional Pressures and Gendered Sexual Scripts. *Qualitative Sociology*, *44*(1), 31–53. https://doi.org/10.1007/s11133-020-09469-6

*Gabbay, N., & Lafontaine, M.-F. (2020). Do Trust and Sexual Intimacy Mediate Attachment’s Pathway Toward Sexual Violence Occurring in Same Sex Romantic Relationships? *Journal of Interpersonal Violence*, *35*(21/22), 5064–5084. https://doi.org/10.1177/0886260517716940

Gaspar, M., Skakoon-Sparling, S., Adam, B. D., Brennan, D. J., Lachowsky, N. J., Cox, J., Moore, D., Hart, T. A., & Grace, D. (2021). “You’re Gay, It’s Just What Happens”: Sexual Minority Men Recounting Experiences of Unwanted Sex in the Era of MeToo. *The Journal of Sex Research*, *58*(9), 1205–1214. https://doi.org/10.1080/00224499.2021.1962236

Gavey, N., Schmidt, J., Braun, V., Fenaughty, J., & Eremin, M. (2009). Unsafe, Unwanted: Sexual Coercion as a Barrier to Safer Sex among Men Who Have Sex with Men. *Journal of Health Psychology*, *14*(7), 1021–1026. https://doi.org/10.1177/1359105309342307

Gilmore, A. K., Koo, K. H., Nguyen, H. V, Granato, H. F., Hughes, T. L., & Kaysen, D. (2014). Sexual assault, drinking norms, and drinking behavior among a national sample of lesbian and bisexual women. *Addictive Behaviors*, *39*(3), 630–636. https://doi.org/10.1016/j.addbeh.2013.11.015

*Gilmore, A. K., Walsh, K., López, C., Fortson, K., Oesterle, D. W., Salamanca, N. K., Orchowski, L. M., & Davis, K. C. (2021). Sexual Assault Victimization: Latinx Identity as a Protective Factor for Sexual Minorities. *Journal of Interpersonal Violence*, *37*(13–14), NP12542–NP12563. https://doi.org/10.1177/0886260521999122

Gold, S. D., Dickstein, B. D., Marx, B. P., & Lexington, J. M. (2009). Psychological Outcomes Among Lesbian Sexual Assault Survivors: An Examination of the Roles of Internalized Homophobia and Experiential Avoidance. *Psychology of Women Quarterly*, *33*(1), 54–66. https://doi.org/10.1111/j.1471-6402.2008.01474.x

Gold, S. D., Marx, B. P., & Lexington, J. M. (2007). Gay male sexual assault survivors: The relations among internalized homophobia, experiential avoidance, and psychological symptom severity. *Behaviour Research & Therapy*, *45*(3), 549–562. https://doi.org/10.1016/j.brat.2006.05.006

*Griner, S. B., Vamos, C. A., Thompson, E. L., Logan, R., Vázquez-Otero, C., & Daley, E. M. (2020). The Intersection of Gender Identity and Violence: Victimization Experienced by Transgender College Students. *Journal of Interpersonal Violence*, *35*(23/24), 5704–5725. https://doi.org/10.1177/0886260517723743

*Gurung, S., Ventuneac, A., Rendina, H. J., Savarese, E., Grov, C., & Parsons, J. T. (2017). Prevalence of Military Sexual Trauma and Sexual Orientation Discrimination Among Lesbian, Gay, Bisexual, and Transgender Military Personnel: A Descriptive Study. *Sexuality Research and Social Policy*, 1. https://doi.org/10.1007/s13178-017-0311-z

Han, S. C., Gallagher, M. W., Franz, M. R., Chen, M. S., Cabral, F. M., & Marx, B. P. (2013). Childhood Sexual Abuse, Alcohol Use, and PTSD Symptoms as Predictors of Adult Sexual Assault Among Lesbians and Gay Men. *Journal of Interpersonal Violence*, *28*(12), 2505–2520. https://doi.org/10.1177/0886260513479030

*Heidt, J. M., Marx, B. P., & Gold, S. D. (2005). Sexual revictimization among sexual minorities: A preliminary study. *Journal of Traumatic Stress*, *18*(5), 533–540. https://doi.org/10.1002/jts.20061z

*Heintz, A. J., & Melendez, R. M. (2006). Intimate Partner Violence and HIV/STD Risk Among Lesbian, Gay, Bisexual, and Transgender Individuals. *Journal of Interpersonal Violence*, *21*(2), 193–208. https://doi.org/10.1177/0886260505282104

Hequembourg, A. L., Bimbi, D., & Parsons, J. T. (2011). Sexual Victimization and Health-Related Indicators Among Sexual Minority Men. *Journal of LGBT Issues in Counseling*, *5*(1), 2–20. https://doi.org/10.1080/15538605.2011.554603

Hequembourg, A. L., Blayney, J. A., Livingston, J. A., Bostwick, W., & Auerbach, S. (2021). A mixed methods investigation of sexual victimisation and coping among sexual minority compared to heterosexual women. *Psychology & Sexuality*, *12*(1/2), 17–36. https://doi.org/10.1080/19419899.2019.1678193

Hequembourg, A. L., Livingston, J. A., & Parks, K. A. (2013). Sexual Victimization and Associated Risks Among Lesbian and Bisexual Women. *Violence Against Women*, *19*(5), 634–657. https://doi.org/10.1177/1077801213490557

*Hequembourg, A., Parks, K., Collins, R. L., & Hughes, T. (2015). Sexual Assault Risks Among Gay and Bisexual Men. *Journal of Sex Research*, *52*(3), 282–295. https://doi.org/10.1080/00224499.2013.856836

Hershow, R. B., Miller, W. C., Giang, L. M., Sripaipan, T., Bhadra, M., Nguyen, S. M., Vu, V. D., Bui, Q., Ha, T. V., & Go, V. F. (2021). Minority Stress and Experience of Sexual Violence Among Men Who Have Sex With Men in Hanoi, Vietnam: Results From a Cross-Sectional Study. *Journal of Interpersonal Violence*, *36*(13/14), 6531–6549. https://doi.org/10.1177/0886260518819884

*Hickson, F. C. I., & Davies, P. M. (1994). Gay men as victims of nonconsensual sex. *Archives of Sexual Behavior*, *23*(3), 281. https://doi.org/10.1007/BF01541564

*Ho, L. Y., Ehman, A. C., & Gross, A. M. (2021). Gender Roles, Sexual Assertiveness, and Sexual Victimization in LGBTQ Individuals. *Sexuality & Culture*, *25*(4), 1469–1489. https://doi.org/10.1007/s12119-021-09819-8

Holmes, S. C., DaFonseca, A. M., & Johnson, D. M. (2021). Sexual Victimization and Disordered Eating in Bisexual Women: A Test of Objectification Theory. *Violence Against Women*, *27*(11), 2021–2042. https://doi.org/10.1177/1077801220963902

*Hughes, T. L., Johnson, T., & Wilsnack, S. C. (2001). Sexual assault and alcohol abuse: a comparison of lesbians and heterosexual women. *Journal of Substance Abuse*, *13*(4), 515–532. https://doi.org/10.1016/s0899-3289(01)00095-5

Hughes, T. L., Szalacha, L. A., Johnson, T. P., Kinnison, K. E., Wilsnack, S. C., & Cho, Y. (2010). Sexual victimization and hazardous drinking among heterosexual and sexual minority women. *Addictive Behaviors*, *35*(12), 1152–1156. https://doi.org/10.1016/j.addbeh.2010.07.004

Jackson, M., Valentine, S., Woodward, E., & Pantalone, D. (2017). Secondary Victimization of Sexual Minority Men Following Disclosure of Sexual Assault: “Victimizing Me All Over Again...” *Sexuality Research & Social Policy: Journal of NSRC*, *14*(3), 275–288. https://doi.org/10.1007/s13178-016-0249-6

Jaffe, A. E., Blayney, J. A., Lewis, M. A., & Kaysen, D. (2020). Prospective Risk for Incapacitated Rape Among Sexual Minority Women: Hookups and Drinking. *Journal of Sex Research*, *57*(7), 922–932. https://doi.org/10.1080/00224499.2019.1661949

*Jenness, V., & Sexton, L. (2021). The centrality of relationships in context: a comparison of factors that predict the sexual and non-sexual victimization of transgender women in prisons for men. *Journal of Crime and Justice*, *45*(3), 259–269. https://doi.org/10.1080/0735648X.2021.1935298

*Jenness, V., Sexton, L., & Sumner, J. (2019). Sexual victimization against transgender women in prison: Consent and coercion in context. *Criminology*, *57*(4), 603–631. https://doi.org/10.1111/1745-9125.12221

Jessie, V. F., & Andréa, B. (2020). “A Situation Where There Aren’t Rules”: Unwanted Sex for Gay, Bisexual, and Questioning Men. *Sociological Science*, *7*(3), 57–74. https://doi.org/10.15195/v7.a3

*Johnson, L. M., Matthews, T. L., & Napper, S. L. (2016). Sexual orientation and sexual assault victimization among US college students. *Social Science Journal*, *53*(2), 174–183. https://doi.org/10.1016/j.soscij.2016.02.007

Kaighobadi, F., Collier, K. L., Reddy, V., Lane, T., & Sandfort, T. G. M. (2020). Sexual violence experiences among black gay, bisexual, and other men who have sex with men and transgender women in South African townships: contributing factors and implications for health. *South African Journal of Psychology*, *50*(2), 170–182. https://doi.org/10.1177/0081246319859449

Kalichman, S. C., Benotsch, E., Rompa, D., Gore-Felton, C., Austin, J., Luke, W., DiFonzo, K., Buckles, J., Kyomugisha, F., & Simpson, D. (2001). Unwanted Sexual Experiences and Sexual Risks in Gay and Bisexual Men: Associations Among Revictimization, Substance Use, and Psychiatric Symptoms. *Journal of Sex Research*, *38*(1), 1–9. https://doi.org/10.1080/00224490109552065

Kalichman, S. C., & Rompa, D. (1995). Sexually Coerced and Noncoerced Gay and Bisexual Men: Factors Relevant to Risk for Human Immunodeficiency Virus (HIV) Infection. *Journal of Sex Research*, *32*(1), 45–50. https://www.jstor.org/stable/3813096

Kammer-Kerwick, M., Wang, A., McClain, T., Hoefer, S., Swartout, K. M., Backes, B., & Busch-Armendariz, N. (2019). Sexual Violence Among Gender and Sexual Minority College Students: The Risk and Extent of Victimization and Related Health and Educational Outcomes. *Journal of Interpersonal Violence*, 886260519883866. https://doi.org/10.1177/0886260519883866

Kelley, M. L., Ehlke, S. J., Braitman, A. L., & Stamates, A. L. (2018). Testing a Model of Binegativity, Drinking-to-Cope Motives, Alcohol Use, and Sexual Coercion Among Self-Identified Bisexual Women. *Journal of Bisexuality*, *18*(4), 478–496. https://doi.org/10.1080/15299716.2018.1481482

Kelley, M. L., Ehlke, S. J., Lewis, R. J., Braitman, A. L., Bostwick, W., Heron, K. E., & Lau-Barraco, C. (2018). Sexual Coercion, Drinking to Cope Motives, and Alcohol-Related Consequences among Self-Identified Bisexual Women. *Substance Use & Misuse*, *53*(7), 1146–1157. https://doi.org/10.1080/10826084.2017.1400565

*Krahé, B., & Berger, A. (2013). Men and women as perpetrators and victims of sexual aggression in heterosexual and same-sex encounters: a study of first-year college students in Germany. *Aggressive Behavior*, *39*(5), 391–404. https://doi.org/10.1002/ab.21482

*Krahé, B., Scheinberger-Olwig, R., & Schütze, S. (2001). Risk Factors of Sexual Aggression and Victimization Among Homosexual Men. *Journal of Applied Social Psychology*, *31*(7), 1385–1408. https://doi.org/10.1111/j.1559-1816.2001.tb02679.x

*Krahé, B., Schütze, S., Fritsche, I., & Waizenhöfer, E. (2000). The Prevalence of Sexual Aggression and Victimization Among Homosexual Men. *Journal of Sex Research*, *37*(2), 142–150. https://www.jstor.org/stable/3813599

Lehavot, K., Molina, Y., & Simoni, J. (2012). Childhood Trauma, Adult Sexual Assault, and Adult Gender Expression among Lesbian and Bisexual Women. *Sex Roles*, *67*(5–6), 272–284. https://doi.org/10.1007/s11199-012-0171-1

Long, S. M., Ullman, S. E., Long, L. M., Mason, G. E., & Starzynski, L. L. (2007). Women’s experiences of male-perpetrated sexual assault by sexual orientation. *Violence & Victims*, *22*(6), 684–701. https://doi.org/10.1891/088667007782793138

*López, G., & Yeater, E. A. (2021). Comparisons of Sexual Victimization Experiences among Sexual Minority and Heterosexual Women. *Journal of Interpersonal Violence*, *36*(7/8), NP4250–NP4270. https://doi.org/10.1177/0886260518787202

*Lucas, C. L., Goldbach, J. T., Mamey, M. R., Kintzle, S., & Castro, C. A. (2018). Military Sexual Assault as a Mediator of the Association Between Posttraumatic Stress Disorder and Depression Among Lesbian, Gay, and Bisexual Veterans. *Journal of Traumatic Stress*, *31*(4), 613–619. https://doi.org/10.1002/jts.22308

Martin, S. L., Fisher, B. S., Warner, T. D., Krebs, C. P., & Lindquist, C. H. (2011). Women’s Sexual Orientations and Their Experiences of Sexual Assault Before and During University. *Women’s Health Issues*, *21*(3), 199–205. https://doi.org/10.1016/j.whi.2010.12.002

*Martin-Storey, A., Paquette, G., Bergeron, M., Dion, J., Daigneault, I., Hébert, M., & Ricci, S. (2018). Sexual Violence on Campus: Differences Across Gender and Sexual Minority Status. *Journal of Adolescent Health*, *62*(6), 701–707. https://doi.org/10.1016/j.jadohealth.2017.12.013

Matsuzaka, S., & Koch, D. E. (2019). Trans Feminine Sexual Violence Experiences: The Intersection of Transphobia and Misogyny. *Affilia: Journal of Women & Social Work*, *34*(1), 28–47. https://doi.org/10.1177/0886109918790929

McGraw, L. K., Tyler, K. A., & Simons, L. G. (2021). Risk Factors for Sexual Assault of Heterosexual and Sexual Minority College Women. *Journal of Interpersonal Violence*. https://doi.org/10.1177/0886260520976224

McKie, R. M., Skakoon-Sparling, S., Levere, D., Sezlik, S., & Humphreys, T. P. (2020). Is There Space for Our Stories? An Examination of North American and Western European Gay, Bi, and Other Men Who Have Sex with Men’s Non-consensual Sexual Experiences. *Journal of Sex Research*, *57*(8), 1014–1025. https://doi.org/10.1080/00224499.2020.1767023

Moschella, E. A., Potter, S. J., & Moynihan, M. M. (2020). Disclosure of Sexual Violence Victimization and Anticipated Social Reactions among Lesbian, Gay, and Bisexual Community College Students. *Journal of Bisexuality*, *20*(1), 66–85. https://doi.org/10.1080/15299716.2020.1715910

*Murchison, G., Boyd, M., & Pachankis, J. (2017). Minority Stress and the Risk of Unwanted Sexual Experiences in LGBQ Undergraduates. *Sex Roles*, *77*(3–4), 221–238. https://doi.org/10.1007/s11199-016-0710-2

Namaste, V., Gaspar, M., Lavoie, S., McClelland, A., Sims, E., Tigchelaar, A., Dietzel, C., & Drummond, J. D. (2020). Willed ambiguity: An exploratory study of sexual misconduct affecting sexual minority male university students in Canada. *Sexualities*, *24*(8). https://doi.org/10.1177/1363460720947308

Nightingale, S. D. (2021). Campus Climate and the Sexual Assault Victimization of LGBQ College Students. *Journal of Homosexuality*, 1–17. https://doi.org/10.1080/00918369.2021.1938466

*Noack-Lundberg, K., Liamputtong, P., Marjadi, B., Ussher, J., Perz, J., Schmied, V., Dune, T., & Brook, E. (2020). Sexual violence and safety: the narratives of transwomen in online forums. *Culture, Health & Sexuality*, *22*(6), 646–659. https://doi.org/10.1080/13691058.2019.1627420

*Palmer, J. E., Williams, E., & Mennicke, A. (2021). Interpersonal Violence Experiences and Disclosure Patterns for Lesbian, Gay, Bisexual, Queer+, and Heterosexual University Students. *Journal of Family Violence*, 1–15. https://doi.org/10.1007/s10896-021-00268-3

*Paquette, G., Martin-Storey, A., Bergeron, M., Dion, J., Daigneault, I., Hébert, M., Ricci, S., & Castonguay-Khounsombath, S. (2019). Trauma Symptoms Resulting From Sexual Violence Among Undergraduate Students: Differences Across Gender and Sexual Minority Status. *Journal of Interpersonal Violence*, 886260519853398. https://doi.org/10.1177/0886260519853398

*Peitzmeier, S. M., Yasin, F., Stephenson, R., Wirtz, A. L., Delegchoimbol, A., Dorjgotov, M., & Baral, S. (2015). Sexual Violence against Men Who Have Sex with Men and Transgender Women in Mongolia: A Mixed-Methods Study of Scope and Consequences. *PLoS ONE*, *10*(10), 1–19. https://doi.org/10.1371/journal.pone.0139320

*Potter, S., Moschella, E., Moynihan, M. M., & Smith, D. (2020). Sexual Violence among LGBQ Community College Students: A Comparison with Their Heterosexual Peers. *Community College Journal of Research and Practice*, *44*(10–12), 787–803. https://doi.org/10.1080/10668926.2019.1706668

*Ratkalkar, M., & Atkin-Plunk, C. A. (2020). Can I Ask for Help? The Relationship Among Incarcerated Males’ Sexual Orientation, Sexual Abuse History, and Perceptions of Rape in Prison. *Journal of Interpersonal Violence*, *35*(19/20), 4117–4140. https://doi.org/10.1177/088626051771444

Ray, C. M., Tyler, K. A., & Gordon Simons, L. (2021). Risk Factors for Forced, Incapacitated, and Coercive Sexual Victimization Among Sexual Minority and Heterosexual Male and Female College Students. *Journal of Interpersonal Violence*, *36*(5/6), 2241–2261. https://doi.org/10.1177/088626051875833

Rhew, I. C., Stappenbeck, C. A., Bedard-Gilligan, M., Hughes, T., & Kaysen, D. (2017). Effects of Sexual Assault on Alcohol Use and Consequences Among Young Adult Sexual Minority Women. *Journal of Consulting & Clinical Psychology*, *85*(5), 424–433. https://doi.org/10.1037/ccp0000202

Richardson, H. B., Armstrong, J. L., Hines, D. A., & Palm Reed, K. M. (2015). Sexual Violence and Help-Seeking Among LGBQ and Heterosexual College Students. *Partner Abuse*, *6*(1), 29–46. https://doi.org/10.1891/1946-6560.6.1.29

Sabidó, M., Kerr, L. R. F. S., Mota, R. S., Benzaken, A. S., de A. Pinho, A., Guimaraes, M. D. C., Dourado, I., Merchan-Hamman, E., & Kendall, C. (2015). Sexual Violence Against Men Who Have Sex with Men in Brazil: A Respondent-Driven Sampling Survey. *AIDS and Behavior*, *19*(9), 1630. https://doi.org/10.1007/s10461-015-1016-z

Salim, S. R., McConnell, A. A., & Messman-Moore, T. L. (2020). Bisexual Women’s Experiences of Stigma and Verbal Sexual Coercion: The Roles of Internalized Heterosexism and Outness. *Psychology of Women Quarterly*, *44*(3), 362–376. https://doi.org/10.1177/0361684320917391

*Satinsky, S., & Jozkowski, K. (2014). Sexual Coercion and Behavior Among a Sample of Sexual Minority Women. *Women & Health*, *54*(2), 77–93. https://doi.org/10.1080/03630242.2013.876487

*Schuyler, A. C., Klemmer, C., Mamey, M. R., Schrager, S. M., Goldbach, J. T., Holloway, I. W., & Castro, C. A. (2020). Experiences of Sexual Harassment, Stalking, and Sexual Assault During Military Service Among LGBT and Non-LGBT Service Members. *Journal of Traumatic Stress*, *33*(3), 257–266. https://doi.org/10.1002/jts.22506

*Seabrook, R. C., McMahon, S., Duquaine, B. C., Johnson, L., & DeSilva, A. (2018). Sexual Assault Victimization and Perceptions of University Climate Among Bisexual Women. *Journal of Bisexuality*, *18*(4), 425–445. https://doi.org/10.1080/15299716.2018.1485070

*Seelman, K. L. (2015). Unequal Treatment of Transgender Individuals in Domestic Violence and Rape Crisis Programs. *Journal of Social Service Research*, *41*(3), 307–325. https://doi.org/10.1080/01488376.2014.987943

*Semple, S., Chavarin, C., Patterson, T., Stockman, J., Pitpitan, E., Strathdee, S., Goodman-Meza, D., Rangel, G., Torres, K., Semple, S. J., Stockman, J. K., Pitpitan, E. V, Strathdee, S. A., Chavarin, C. V, & Patterson, T. L. (2017). Correlates of Sexual Violence Among Men Who Have Sex With Men in Tijuana, Mexico. *Archives of Sexual Behavior*, *46*(4), 1011–1023. https://doi.org/10.1007/s10508-016-0747-x

*Shaw, S. Y., Lorway, R. R., Deering, K. N., Avery, L., Mohan, H. L., Bhattacharjee, P., Reza-Paul, S., Isac, S., Ramesh, B. M., Washington, R., Moses, S., & Blanchard, J. F. (2012). Factors Associated with Sexual Violence against Men Who Have Sex with Men and Transgendered Individuals in Karnataka, India. *PLoS ONE*, *7*(3), 1–8. https://doi.org/10.1371/journal.pone.0031705

Sigurvinsdottir, R., & Ullman, S. (2016a). Sexual Orientation, Race, and Trauma as Predictors of Sexual Assault Recovery. *Journal of Family Violence*, *31*(7), 913–921. https://doi.org/10.1007/s10896-015-9793-8

*Sigurvinsdottir, R., & Ullman, S. E. (2016b). Sexual Assault in Bisexual and Heterosexual Women Survivors. *Journal of Bisexuality*, *16*(2), 163–180. https://doi.org/10.1080/15299716.2015.1136254

*Sigurvinsdottir, R., Ullman, S. E., & Ulman, S. E. (2015). The Role of Sexual Orientation in the Victimization and Recovery of Sexual Assault Survivors. *Violence & Victims*, *30*(4), 636–648. https://doi.org/10.1891/0886-6708.VV-D-13-00066.

Smidt, A. M., Rosenthal, M. N., Smith, C. P., & Freyd, J. J. (2021). Out and in Harm’s Way: Sexual Minority Students’ Psychological and Physical Health after Institutional Betrayal and Sexual Assault. *Journal of Child Sexual Abuse*, *30*(1), 41–55. https://doi.org/10.1080/10538712.2019.1581867

*Snyder, J. A., Scherer, H. L., & Fisher, B. S. (2018). Interpersonal Violence among College Students: Does Sexual Orientation Impact Risk of Victimization? *Journal of School Violence*, *17*(1), 1–15. https://doi.org/https://doi.org/10.1080/15388220.2016.1190934

Solomon, D. T., Combs, E. M., Allen, K., Roles, S., DiCarlo, S., Reed, O., & Klaver, S. J. (2021). The impact of minority stress and gender identity on PTSD outcomes in sexual minority survivors of interpersonal trauma. *Psychology & Sexuality*, *12*(1/2), 64–78. https://doi.org/10.1080/19419899.2019.1690033

*Staples, J. M., & Fuller, C. C. (2021). Adult Sexual Assault Severity among Transgender People of Color: The Impact of Double Marginalization. *Journal of Aggression, Maltreatment & Trauma*, *30*(5), 694–706. https://doi.org/10.1080/10926771.2021.1894291

*Stoddard, J., Dibble, S., & Fineman, N. (2009). Sexual and Physical Abuse: A Comparison Between Lesbians and Their Heterosexual Sisters. *Journal of Homosexuality*, *56*(4), 407–420. https://doi.org/10.1080/00918360902821395

*Strike, C., Myers, T., Calzavara, L., & Haubrich, D. (2001). Sexual coercion among young street-involved adults: perpetrators’ and victims’ perspectives. *Violence and Victims*, *16*(5), 537–551. http://www.ncbi.nlm.nih.gov/pubmed/11688928

*Tilapaugh, D. (2016). Resisting erasure: Critical influences for men who survived sexual violence in higher education. *Social Alternatives*, *35*(3), 11–17. https://doi.org/10.3316/informit.617681441434927

*Tilley, D. S., Kolodetsky, A., Cottrell, D., & Tilton, A. (2020). Correlates to Increased Risk of Sexual Assault and Sexual Harassment Among LGBT+ University Students. *Journal of Forensic Nursing*, *16*(2), 63–72. https://doi.org/10.1097/JFN.0000000000000284

*Toro-Alfonso, J., & Rodríguez-Madera, S. (2004). Sexual Coercion in a Sample of Puerto Rican Gay Males. *Journal of Gay & Lesbian Social Services*, *17*(1), 47–58. https://doi.org/10.1300/J041v17n01_04

*Twinley, R. (2017). Woman-to-woman rape and sexual assault, and its impact upon the occupation of work: Victim/survivors’ life roles of worker or student as disruptive and preservative. *Work*, *56*(4), 505–517. https://doi.org/10.3233/WOR-172529

* Ussher, J. M., Hawkey, A., Perz, J., Liamputtong, P., Sekar, J., Marjadi, B., Schmied, V., Dune, T., & Brook, E. (2020). Crossing Boundaries and Fetishization: Experiences of Sexual Violence for Trans Women of Color. *Journal of Interpersonal Violence*, 1. https://doi.org/10.1177/0886260520949149

*VanderLaan, D. P., & Vasey, P. L. (2009). Patterns of Sexual Coercion in Heterosexual and Non-Heterosexual Men and Women. *Archives of Sexual Behavior*, *38*(6), 987–999. https://doi.org/10.1007/s10508-009-9480-z

*Waldner-Haugrud, L. K., & Gratch, L. V. (1997). Sexual Coercion in Gay/Lesbian Relationships: Descriptives and Gender Differences. *Violence & Victims*, *12*(1), 87–98.

*Watson, L. B., Craney, R. S., Greenwalt, S. K., Beaumont, M., Whitney, C., & Flores, M. J. (2021). “I Was a Game or a Fetish Object”: Diverse Bisexual Women’s Sexual Assault Experiences and Effects on Bisexual Identity. *Journal of Bisexuality*, *21*(2), 225–261. https://doi.org/10.1080/15299716.2021.1932008

*Wells, B. E., Starks, T. J., Robel, E., Kelly, B. C., Parsons, J. T., & Golub, S. A. (2016). From Sexual Assault to Sexual Risk. *Journal of Interpersonal Violence*, *31*(20), 3377–3395. https://doi.org/10.1177/0886260515584353

Wilkerson, J. M., Di Paola, A., Nieto, D., Schick, V., Latini, D. M., Braun-Harvey, D., Zoschke, I. N., & McCurdy, S. (2021). Sexual Violence and Chemsex among Substance-Using Sexual and Gender Minorities in Texas. *Substance Use & Misuse*, 1–10. https://doi.org/10.1080/10826084.2021.1975743

*Wilson, M., Simpson, P. L., Butler, T. G., Yap, L., Richters, J., & Donovan, B. (2017). ‘You’re a woman, a convenience, a cat, a poof, a thing, an idiot’: Transgender women negotiating sexual experiences in men’s prisons in Australia. *Sexualities*, *20*(3), 380–402. https://search.ebscohost.com/login.aspx?direct=true&AuthType=sso&db=sih&AN=121217325&site=eds-live&scope=site&custid=s2775460
