# Supplementary material for: LGBTQ+ Adult Sexual Violence Critical Scoping Review: Insights into Victimization and Perpetration
Source: Trauma Violence Abuse. 2025 Jan 22;27(2):305–18. doi: 10.1177/15248380241311928 (PMC12953667; doi:10.1177/15248380241311928)
Supplement: sj-docx-2-tva-10.1177_15248380241311928 – Supplemental material for LGBTQ+ Adult Sexual Violence Critical Scoping Review: Insights into Victimization and Perpetration [file sj-docx-2-tva-10.1177_15248380241311928.docx]

**Appendix B - Summary of Research Studies**

| Author/Year | Title and journal | Country | Aim/purpose | Methods | Sample |
| --- | --- | --- | --- | --- | --- |
| Aspin, Reynolds, Lehavot & Taiapa (2009) | An investigation of the phenomenon of non-consensual sex among Māori men who have sex with men | Australia | To develop an understanding of non-consensual sex amongst Māori MSM. | - Qualitative  - Interviews | N = 8  LGBQ+ men |
| Backhaus, Lipson, Fisher, Kawachi & Pedrelli (2019) | Sexual assault, sense of belonging, depression and suicidality among LGBQ and heterosexual college students | United States | To compare the effect of sexual orientation, history of sexual assault and sense of belonging on depression and suicidality college students. | - Qualitative  - Cross-sectional survey | N = 60194  LGBQ+ women; LGBQ+ men; Intersex people; Heterosexual women; Heterosexual men; |
| Balsam, Lehavot & Beadnell (2011) | Sexual revictimization and mental health: A comparison of lesbians, gay men, and heterosexual women | United States | To compare adult lesbians, gay men, and heterosexual women on prevalence and mental health correlates of sexual revictimization | - Quantitative  - Survey | N = 871  LGBQ+ women; LGBQ+ men; Heterosexual women |
| Beckman, Shipherd, Simpson & Lehavot (2018) | Military sexual assault in transgender veterans: Results from a nationwide survey | United States | - To identify prevalence of military sexual assault and to assess its association with other factors.  - Evaluated association between military sexual assault and mental health. | - Quantitative  - Survey | N = 221  Transgender people |
| Bedera, & Nordmeyer (2020) | An inherently masculine practice: Understanding the sexual victimization of queer women | United States | - Investigate LGBQ+ women’s sexual violence  - Gain information on the perpetrator | - Qualitative  - Interviews | N = 40  LGBQ+ women |
| Braun, Terry, Gavey & Fenaughty (2009) | ‘Risk’ and sexual coercion among gay and bisexual men in Aotearoa/New Zealand - key informant accounts | New Zealand | - To examine the concept of 'risk' through qualitative interviews with gay and bisexual men. | - Qualitative  - Interviews | N = 23  LGBQ+ men |
| Braun, Schmidt, Gavey & Fenaughty, (2009) | Sexual coercion among gay and bisexual men in Aotearoa/New Zealand | New Zealand | - To report on interviews with gay and bisexual men about their experiences of forced, coerced, or unwanted sex | - Qualitative  - Interviews | N = 19 LGBQ+ men |
| Connolly, Aldridge, Davies, Maier, Ferris, Gilchrist, & Winstock (2021) | Comparing transgender and cisgender experiences of being taken advantage of sexually while under the influence of alcohol and/or other drugs | International survey, respondents predominantly from Europe, United States & Australia. | - To compare trans and cis peoples experiences of sexual violence respondents on lifetime and last year experiences with sexual violence while under the influence  - To compare trans participants lifetime and last year experiences with sexual violence while under the influence | - Quantitative  - Survey | N = 75,413  LGBQ+ women; LGBQ+ men; Transgender people; Heterosexual women; Heterosexual men; |
| Cook-Daniels & munson (2010) | Sexual violence, elder abuse, and sexuality of transgender adults, age 50+: results of three surveys | United States | - To report on the findings of three online surveys of transgender adults age 50 years and older on three topics: sexual violence, elder abuse, and sexuality. | - Mixed  - Survey  - Interviews | N = 302  Transgender people |
| Coulter, Mair, Miller, Blosnich, Matthews, McCauley, Coulter, Blosnich, Matthews & McCauley (2017) | Prevalence of past-year sexual assault victimization among undergraduate students: Exploring differences by and intersections of gender identity, sexual identity, and race/ethnicity | United States | - To explore the effects of sexual identity, gender identity, and race/ethnicity on past-year sexual assault among US college students. | - Quantitative  - Survey | N = 71,421  LGBQ+ women; LGBQ+ men; Transgender people; Heterosexual women; Heterosexual men; |
| Coulter & Rankin, (2020) | College sexual assault and campus climate for sexual- and gender-minority undergraduate students | United States | - To assess whether campus climate and perceived inclusion of sexual and gender minority people has an impact on sexual and gender minority experiences of college sexual assault | - Quantitative  - Cross-sectional surveys | N = 1,925  LGBQ+ women; LGBQ+ men; Transgender people |
| Crump & Byers (2017) | Sexual well-being of sexual minority women in dating relationships who have experienced childhood sexual abuse and/or adolescent and adult sexual victimization | Canada | - To examine the impact of CSA and adult sexual assault on sexual wellbeing in sexual minority women. | - Quantitative  - Survey | N = 299  LGBQ+ women |
| DeLaney, Williams, Mosley, Haw & Dick (2020) | The associations between sexual victimization and health outcomes among LGBQA college students: Examining the moderating role of social support | United States | - To understand how sexual victimization influences mental health and substance use outcomes for LGBQA emerging adults in college. | - Quantitative  - Survey, yearly while enrolled | N = 234  LGBQ+ women; LGBQ+ men |
| Drückler, Speulman, van Rooijen & De Vries (2021) | Sexual consent and chemsex: A quantitative study on sexualised drug use and non-consensual sex among men who have sex with men in Amsterdam, the Netherlands | Netherland | - To assess whether non-consensual sex is associated with chemsex amongst MSM. | - Quantitative  - Survey | N = 891  LGBQ+ men |
| Eisenberg, Lust, Mathiason & Porta (2021) | Sexual assault, sexual orientation, and reporting among college students | United States | - To explore whether LGBQ students report assaults at similar rates to heterosexual students. | - Quantitative  - Survey | N = 10,646  LGBQ+ women; LGBQ+ men; Heterosexual women; Heterosexual men |
| Fernández-Rouco, Fernández-Fuertes, Carcedo, Lázaro-Visa, & Gómez-Pérez (2017) | Sexual violence history and welfare in transgender people | Spain | To examine experiences of sexual violence and its impacts on Spanish trans people. | - Qualitative  - Cross-sectional interviews | N = 33  Transgender people |
| Fileborn (2014) | Accounting for space, place and identity: GLBTIQ young adults’ experiences and understandings of unwanted sexual attention in clubs and pubs | Australia | To understand young GLBTIQ people's experiences of unwanted sexual attention in licensed venues. | - Mixed  - Surveys  - Focus groups  - Interviews | N = 78  LGBQ+ women; LGBQ+ men |
| Flanders, Anderson, Tarasoff, & Robinson (2019) | Bisexual stigma, sexual violence, and sexual health among bisexual and other plurisexual women: A cross-sectional survey study | United States and Canada | - To analyze the relationship between bisexual-specific stigma and sexual violence, as well as other sexual health outcomes. | - Quantitative  - Cross-sectional survey | N = 323  LGBQ+ women; Transgender people |
| Flanders, Anderson, & Tarasoff (2020) | Young bisexual people's experiences of sexual violence: A mixed-methods study | United States and Canada | - To investigate young bisexual people’s experiences of sexual violence.  - To asses the relationship between bisexual stigma and sexual violence outcomes | - Mixed  - Cross-sectional survey and interviews | N = 245  LGBQ+ women; LGBQ+ men; Transgender people |
| Fontanesi, D'Urso, Panzeri & Pace (2020) | The role of attachment style in predicting emotional abuse and sexual coercion in gay and lesbian people: An explorative study | Italy | To investigate whether there is a relationship between attachment style and perpetrating sexual coercion in a sample of people who are gay and lesbian. | - Quantitative  - Survey | N = 182  LGBQ+ women; LGBQ+ men |
| Ford (2019) | Unwanted sex on campus: The overlooked role of interactional pressures and gendered sexual scripts | United States | To examine heterosexual and queer college students accounts of unwanted sex. | - Qualitative  - Interviews | N = 110  LGBQ+ women; LGBQ+ men; Heterosexual women; Heterosexual men |
| Gabbay & Lafontaine (2020) | Do trust and sexual intimacy mediate attachment's pathway toward sexual violence occurring in same sex romantic relationships? | Canada | To examine the role of romantic attachment and perpetrated sexual violence in same sex intimate partners. | - Quantitative  - Survey | N = 310  LGBQ+ women; LGBQ+ men |
| Gaspar, Skakoon-Sparling, Adam, Brennan, Lachowsky, Cox, Moore, Hart, & Grace (2021) | "You're gay, it's just what happens": Sexual minority men recounting experiences of unwanted sex in the era of MeToo | Canada | To understand experiences of sexual coercion among gay, bisexual, queer, and other men. | - Qualitative  - Interviews | N = 24  LGBQ+ men; Transgender people |
| Gavey, Schmidt, Braun, Fenaughty, Eremin (2009) | Unsafe, unwanted: Sexual coercion as a barrier to safer sex among men who have sex with men | New Zealand | To understand how dynamics of sexual coercion compromise gay and bisexual men's ability to practice safe sex. | - Qualitative  - Interviews  - Focus groups | N = 63  LGBQ+ men |
| Gilmore, Koo, Nguyen, Granato, Hughes, & Kaysen (2014) | Sexual assault, drinking norms, and drinking behavior among a national sample of lesbian and bisexual women | United States | - To examine the relationship among sexual assault history, drinking behavior, and drinking norms in emerging adult lesbian and bisexual women. | - Quantitative  - Survey | N = 1,094  LGBQ+ women |
| Gilmore, Walsh, López, Fortson, Oesterle, Salamanca, Orchowski, & Davis (2021) | Sexual assault victimization: Latinx identity as a protective factor for sexual minorities | United States | - To understand experiences of sexual assault victimization among students who identify as an ethnic minority and a sexual minority.  - To understand the role of alcohol in these assaults. | - Quantitative  - Survey | N = 506  LGBQ+ women; LGBQ+ men; Transgender people; Heterosexual women; Heterosexual men |
| Gold, Marx & Lexington (2007) | Gay male sexual assault survivors: The relations among internalized homophobia, experiential avoidance, and psychological symptom severity | United States | - To explore internalized homophobia, experiential avoidance, and psychological symptom severity in gay male sexual assault survivors | - Quantitative  - Survey | N = 74  LGBQ+ men |
| Gold, Dickstein, Marx & Lexington (2009) | Psychological outcomes among lesbian sexual assault survivors: An examination of the roles of internalized homophobia and experiential avoidance | United States | - To assess how CSA and/or adult sexual abuse experienced by lesbians can impact PTSD, internalised homophobia and avoidant behaviour | - Quantitative  - Survey | N = 72  LGBQ+ women |
| Griner, Vamos, Thompson, Logan, Vá¡zquez-Otero, & Daley (2020) | The intersection of gender identity and violence: Victimization experienced by transgender college students | United States | - To explore the rates of violence experienced by transgender students compared with cisgender students. | - Quantitative  - Survey | n = 88,975  LGBQ+ women; LGBQ+ men; Transgender people; Heterosexual women; Heterosexual men; |
| Gurung, Ventuneac, Rendina, Savarese, Grov & Parsons (2017) | Prevalence of military sexual trauma and sexual orientation discrimination among lesbian, gay, bisexual, and transgender military personnel: A descriptive study | United States | - To assess the prevalence of sexual orientation discrimination and sexual harassment and sexual assault experienced by LGBT people in the US military. | - Quantitative  - Survey | N = 253  LGBQ+ women; LGBQ+ men; Transgender people |
| Han, Gallagher, Franz, Chen, Cabral, & Marx (2013) | Childhood sexual abuse, alcohol use, and PTSD symptoms as predictors of adult sexual assault among lesbians and gay men | United States | - To examine the extent to which CSA, alcohol use, and PTSD symptoms related to adult sexual assault among lesbians and gay men. | - Quantitative  - Survey | N = 239  LGBQ+ women; LGBQ+ men |
| Heidt, Marx & Gold (2005) | Sexual revictimization among sexual minorities: A preliminary study | United States | - To examine patterns of sexual assault and its psychological correlates among gay men, lesbians, and bisexual men and women. | - Quantitative  - Survey | N = 307  LGBQ+ women; LGBQ+ men |
| Heintz, & Melendez (2006) | Intimate partner Violence and HIV/STD risk among lesbian, gay, bisexual, and transgender individuals | United States | - To examine if sexual violence in the relationship further affected safer sex negotiation. | - Quantitative  - Survey | N = 58  LGBQ+ women; LGBQ+ men; Transgender people |
| Hequembourg, Bimbi & Parsons (2011) | Sexual victimization and health-related indicators among sexual minority men | United States | - To report rates of CSA and adult sexual victimization among a community sample of GB men  - To examine how sexual victimization histories impact health-related outcomes | - Quantitative  - Cross-sectional  - Street-intercept survey | N = 634  LGBQ+ men |
| Hequembourg, Livingston, & Parks (2013) | Sexual victimization and associated risks among lesbian and bisexual women | United States | - To examine relationship among CSA, risky alcohol use and adult sexual violence among LB women | - Mixed  - Survey  - Interview | N = 205  LGBQ+ women |
| Hequembourg, Parks, Collins & Hughes (2015) | Sexual assault risks among gay and bisexual men | United States | - To examine lifetime patterns of sexual assault and associated risks among GB men. | - Mixed  - Survey  - Interviews | N = 183  LGBQ+ men |
| Hequembourg, Blayney, Livingston, Bostwick & Auerbach (2021) | A mixed-methods investigation of sexual victimisation and coping among sexual minority compared to heterosexual women | United States | - To understand the SV recovery process for sexual minority women, with a particular focus on bisexual women | - Mixed  - Survey  - Interviews | N = 246  LGBQ+ women |
| Hershow, Miller, William, Giang, Sripaipan, Bhadra, Nguyen, Vu, Bui, Ha, & Go (2021) | Minority stress and experience of sexual violence among men who have sex with men in Hanoi, Vietnam: Results from a cross-sectional study | Vietnam | - To examine relationship between homosexuality stigma measures and sexual violence in the last 12 months of MSM in Hanoi | - Quantitative  - Survey | N = 202  LGBQ+ men |
| Hickson & Davies (1994) | Gay Men as Victims of Nonconsensual Sex | UK | - To explore gay men’s first experiences of sexual assault. | - Mixed  - Interviews  - Survey | N = 930  LGBQ+ men |
| Ho, Ehman & Gross (2021) | Gender roles, sexual assertiveness, and sexual victimization in LGBTQ individuals | United States | - To measure the relationship between sexual victimization, gender roles, and sexual assertiveness in an LGBTQ sample. | - Quantitative  - Survey | N = 455  LGBQ+ women; LGBQ+ men; Transgender people |
| Holmes, DaFonseca & Johnson (2021) | Sexual victimization and disordered eating in bisexual women: A test of objectification theory | United States | - To identify the relative rates of sexual violence and disordered eating in heterosexual and bisexual women | - Quantitative  - Survey | N = 499  LGBQ+ women; Heterosexual women |
| Hughes, Johnson & Wilsnack (2001) | Sexual assault and alcohol abuse: A comparison of lesbians and heterosexual women | United States | - To compare lesbian and heterosexual women’s experiences of sexual assault  - To investigate relationships between sexual assault and alcohol abuse | - Quantitative  - Interview Questionnaire | N = 120  LGBQ+ women; Heterosexual women |
| Hughes, Szalacha, Johnson, Kinnison, Wilsnack & Cho (2010) | Sexual victimization and hazardous drinking among heterosexual and sexual minority women | United States | - To examine and compare relationship between sexual victimization and hazardous drinking in heterosexual and sexual minority women | - Quantitative  - Pooled data from two surveys | N = 953  LGBQ+ women; Heterosexual women |
| Jackson, Valentine, Woodward & Pantalone (2017) | Secondary victimization of sexual minority men following disclosure of sexual assault: "victimizing me all over again..." | United States | - To explore secondary victimization of sexual minority men who disclose to a care provider | - Qualitative  - Interviews | N = 18  LGBQ+ men |
| Jaffe, Blayney, Lewis, & Kaysen (2020) | Prospective risk for incapacitated rape among sexual minority women: Hookups and drinking | United States | - To assess whether sexual minority women are at greater risk for heavy drinking and sexual victimisation than heterosexual women | - Quantitative  - Survey | N = 1,057  LGBQ+ women; Heterosexual women |
| Jenness, Sexton & Sumner (2019) | Sexual victimization against transgender women in prison: Consent and coercion in context | United States | - To analyse the factors and processes that shape trans women’s experience of sexual victimization in men’s prison | - Mixed  - Interviews  - Prison data | N = 315  Transgender women |
| Jenness & Sexton (2021) | The centrality of relationships in context: A comparison of factors that predict the sexual and non-sexual victimization of transgender women in prisons for men | United States | - To assess how certain factors shape the probability of transgender women in prisons for men experiencing sexual victimization and non-sexual physical assault | - Qualitative  - Interviews | N = 315  Transgender women |
| Ford & Becker (2020) | "A situation where there aren't rules: Unwanted sex for gay, bisexual, and questioning men | United States | - To interview GBQ men about their experiences of unwanted sex in college | - Qualitative  - Interviews | N = 18  LGBQ+ men |
| Johnson, Matthews & Napper (2016) | Sexual orientation and sexual assault victimization among US college students | United States | - To examine risk of sexual victimization among sexual minority college students compared to heterosexual student | Quantitative | N = 29,295  LGBQ+ women; LGBQ+ men; Transgender people; Heterosexual women; Heterosexual men; |
| Kaighobadi, Collier, Reddy, Lane & Sandfort (2020) | Sexual violence experiences among black gay, bisexual, and other men who have sex with men and transgender women in South African townships: Contributing factors and implications for health | South Africa | - To examine experiences with sexual violence among black African GB, and MSM and transgender women in townships surrounding Pretoria | - Qualitative  - Interviews | N = 81  LGBQ+ men; Transgender women |
| Kalichman & Rompa (1995) | Sexually coerced and noncoerced gay and bisexual men: Factors relevant to risk for human immunodeficiency Virus (HIV) infection | United States | - To study sexual coercion in homosexual relationships and whether it could be a contributing factor in risk for HIV | - Quantitative  - Survey | N = 196  LGBQ+ men |
| Kalichman, Benotsch, Rompa, Gore-Felton, Austin, Luke, DiFonzo, Buckles, Kyomugisha & Simpson (2001) | Unwanted sexual experiences and sexual risks in gay and bisexual men: Association amount revictimisation, substance use, and psychiatric symptoms | United States | - To examine the histories of unwanted sexual contact in childhood and adulthood in relation to HIV risk behaviour, substance use, and mental health issues in MSM. | - Quantitative  - Survey | N = 595  LGBQ+ men |
| Kammer-Kerwick, Wang, McClain, Hoefer, Swartout, Backes & Busch-Armendariz (2019) | Sexual violence among gender and sexual minority college students: The risk and extent of victimization and related health and educational outcomes | United States | - To assess what effects gender identity and sexual orientation have on the risk and extent of sexual violence among students. | - Quantitative  - Survey | N = 17,039  LGBQ+ women; LGBQ+ men; Transgender people |
| Kelley, Ehlke, Braitman & Stamates (2018) | Testing a model of binegativity, drinking-to-cope motives, alcohol use, and sexual coercion among self-identified bisexual women | United States | - To test the associations between binegativity, alcohol use, drinking to cope motivations, and sexual coercion in a sample of bisexual women. | - Quantitative  - Survey | N = 225  LGBQ+ women |
| Kelley, Ehlke, Lewis, Braitman, Bostwick, Heron, & Lau-Barraco (2018) | Sexual coercion, drinking to cope motives, and alcohol-related consequences among self-identified bisexual women | United States | - To test a moderated mediation model in which sexual coercion was hypothesized to be associated with alcohol-related consequences | - Quantitative  - Survey | N = 107  LGBQ+ women |
| Krahé, Schütze, Fritsche & Waizenhöfer (2000) | The prevalence of sexual aggression and victimisation among homosexual men | Germany | - To examine the prevalence of sexual victimisation and perpetration of sexually aggressive acts in a sample of homosexual men. | - Quantitative  - Survey | N = 310  LGBQ+ men |
| Krahé, Scheinberger-Olwig & Schütze, (2001) | Risk factors of sexual aggression and victimization among homosexual men | Germany | - To examine the risk factors of sexual aggression and victimization among homosexual men | - Quantitative  - Survey | N = 310  LGBQ+ men |
| Krahé & Berger (2013) | Men and women as perpetrators and victims of sexual aggression in heterosexual and same-sex encounters: A study of first year college students in Germany | Germany | - To examine the prevalence of sexual aggression and victimization in college students | - Quantitative  - Survey | N = 2,149  LGBQ+ women; LGBQ+ men; Heterosexual women; Heterosexual men |
| Lehavot, Molina & Simoni (2012) | Childhood trauma, adult sexual assault, and adult gender expression among lesbian and bisexual women | United States | - To examine associations between current gender identity with CSA and adult sexual assault in adult sexual minority women. | - Qualitative  - Survey | N = 1,243  LGBQ+ women |
| Long, Ullman, Long, Mason & Starynski (2007) | Women's experiences of male-perpetrated sexual assault by sexual orientation | United States | - To examine differences in male-perpetrated adult sexual assault experiences among women of various sexual orientations using a large urban convenience sample. | - Quantitative  - Survey | N = 1,022  LGBQ+ women; Heterosexual women |
| López & Yeater (2021) | Comparisons of sexual victimization experiences among sexual minority and heterosexual women | United States | - To examine the associations among heterosexual and sexual minority women, sexual victimization experiences, coping strategies, and mental health. | - Mixed  - Quantitative survey  - Qualitative survey | N = 177  LGBQ+ women; Heterosexual women |
| Lucas, Goldbach, Mamey, Kintzle, & Castro (2018) | Military sexual assault as a mediator of the association between posttraumatic stress disorder and depression among lesbian, gay, and bisexual veterans | United States | - To compare military sexual assault, PTSD, and depression in LGB individuals and their non-LGB peers in two community samples of veterans | - Quantitative  - Drawn from two surveys | N = 440  LGBQ+ women; LGBQ+ men |
| Martin, Fisher, Warner, Krebs & Lindquist (2011) | Women's sexual orientations and their experiences of sexual assault before and during university | United States | - To examine relationships between women’s sexual orientations and their sexual assault experiences before and during university | - Quantitative  - Survey | N = 5,239  LGBQ+ women; Heterosexual women |
| Martin-Storey, Paquette, Bergeron, Dion, Daigneault, Hébert & Ricci (2018) | Sexual violence on campus: Differences across gender and sexual minority status | Canada | - To identify vulnerability among sexual and gender minority students with regard to sexual violence  - To explore if the context of this violence differs across sexual and gender minority status. | - Quantitative  - Survey | N = 4,264  LGBQ+ women; LGBQ+ men; Transgender &/or non-binary people |
| Matsuzaka & David (2019) | Trans feminine sexual violence experiences: The intersection of transphobia and misogyny | United States | - To analyze in-depth interviews with trans feminine adults to explore their experiences of sexual victimization. | Qualitative | N = 10  Trans feminine adults |
| McGraw, Tyler & Simons (2020) | Risk factors for sexual assault of heterosexual and sexual minority college women | United States | - To examine protective factors and risk factors sexual assault and whether they vary by sexual orientation among college women. | - Quantitative  - | N = 1,482  LGBQ+ women; Heterosexual women |
| McKie, Skakoon-Sparling, Levere, Sezlik & Humphreys (2020) | Is there space for our stories? An examination of North American and western European gay, bi, and other men who have sex with men’s non-consensual sexual experiences | North American and western European | - To examine the understanding and behaviors relating to sexual consent among GB and other MSM internationally. | - Mixed  - Qualitative and quantitative survey | N = 350  LGBQ+ men |
| Moschella, Potter & Moynihan (2020) | Disclosure of sexual violence victimization and anticipated social reactions among lesbian, gay, and bisexual community college students | United States | - To compare rates of disclosure and anticipated social reactions of LGB and heterosexual community college students who reported at least one incident of sexual violence victimization since enrolling. | - Quantitative  - Survey | N = 677  LGBQ+ women; LGBQ+ men; Heterosexual women; Heterosexual men |
| Murchison, Boyd & Pachankis (2017) | Minority stress and the risk of unwanted sexual experiences in LGBQ undergraduates | United States | - To determine whether minority stress predicted unwanted sexual experiences among LGBQ undergraduates.  - Assess whether sense of LGBTQ community was a protective factor. | - Quantitative  - Survey | N = 763  LGBQ+ women; LGBQ+ men; Transgender people; Non-binary people |
| Namaste, Gaspar, Lavoie, McClelland, Sims, Tigchelaar, Dietzel & Drummond (2021) | Willed ambiguity: An exploratory study of sexual misconduct affecting sexual minority male university students in Canada | Canada | - To reflect on sexual misconduct affecting male students by males in positions of authority in the university context. | - Qualitative  - Interviews | N = 13  LGBQ+ men |
| Nightingale (2021) | Campus climate and the sexual assault victimization of LGBQ college students | United States | - To explore the relationship of campus climate with sexual assault victimization for sexual minority college students | - Quantitative  - Survey | N = 1,110  LGBQ+ women; LGBQ+ men |
| Noack-Lundberg, Liamputtong, Marjadi, Ussher, Perz, Schmied, Dune & Brook (2020) | Sexual violence and safety: the narratives of trans women in online forums | Australia | - To analyse an online forum to explore experiences of sexual violence and safety among transwomen, with a particular focus on transwomen of colour. | - Qualitative  - Online forum analysis | Transwomen |
| Palmer, Williams & Mennicke (2021) | Interpersonal violence experiences and disclosure patterns for lesbian, gay, bisexual, queer+, and heterosexual university students | United States | - To compare LGBQ+ to heterosexual student experiences with victimization (intimate partner and sexual violence) and disclosure. | - Quantitative  - Survey | N = 1,582  LGBQ+ women; LGBQ+ men; Transgender & non-binary people; Heterosexual women; Heterosexual men |
| Paquette, Martin-Storey, Bergeron, Dion, Daigneault, Hébert, Ricci, Castonguay-Khounsombath (2019) | Trauma symptoms resulting from sexual violence among undergraduate students: Differences across gender and sexual minority status | Canada | - To determine if, among university students who have experienced one or more acts of sexual violence, gender and sexual minority students were more likely to report trauma symptoms and whether two contextual factors were associated. | - Quantitative  - Survey | N = 1,196  LGBQ+ women; LGBQ+ men; Transgender and non-binary people; Heterosexual women; Heterosexual men; |
| Peitzmeier, Yasin, Stephenson, Wirtz, Delegchoimbol, Dorjgotov & Baral, (2015) | Sexual violence against men who have sex with men and transgender women in Mongolia: a mixed-methods study of scope and consequences | Mongolia | - To understand the prevalence, correlates and consequences of sexual violence for MSM and transgender women in Mongolia | - Mixed  - Survey  - Interviews  - Focus groups | N = 313  LGBQ+ men; Transgender women |
| Potter, Moschella, Moynihan & Smith (2020) | Sexual violence among LGBQ community college students: A comparison with their heterosexual peers | United States | - To compare rates of sexual violence between LGBQ students and their non-LGBQ counterparts using data from a campus climate survey administered at seven community colleges | - Quantitative  - Survey | N = 806  LGBQ+ women; LGBQ+ men; Transgender people; Heterosexual women; Heterosexual men |
| Ratkalkar & Atkin-Plunk (2020) | Can I ask for help? The relationship among incarcerated males’ sexual orientation, sexual abuse history, and perceptions of rape in prison | United States | - To examine the perceived fear of rape and mental health treatment-seeking behavior in two vulnerable prison populations: gay and bisexual inmates and inmates with CSA history | - Qualitative  - Interview | N = 409  LGBQ+ men |
| Ray, Tyler & Simons (2021) | Risk factors for forced, incapacitated, and coercive sexual victimization among sexual minority and heterosexual male and female college students | United States | - To examine whether risk factors for three types of sexual victimization (i.e., forced, incapacitated, and coerced) differ by gender and sexual orientation among college students. | - Quantitative  - Survey | N = 1,143  LGBQ+ women; LGBQ+ men |
| Rhew, Stappenbeck, Bedard-Gilligan, Hughes & Kaysen (2017) | Effects of sexual assault on alcohol use and consequences among young adult sexual minority women | United States | - To examine the effects of sexual assault victimization on later typical alcohol use and alcohol-related consequences among young sexual minority women. | - Quantitative  - Survey | N = 1,057  LGBQ+ women |
| Richardson, Armstrong, Hines & Palm Reed (2015) | Sexual violence and help-seeking among LGBQ and heterosexual college students | United States | - To examine reports of sexual violence, substance use, and help-seeking events among LGBQ and heterosexual college students. | - Quantitative  - Survey | N = 2,790  LGBQ+ women; LGBQ+ men; Heterosexual women; Heterosexual men |
| Sabidó, Kerr, Mota, Benzaken, de A. Pinho, Guimaraes, Dourado, Merchan-Hamman & Kendall (2015) | Sexual violence against men who have sex with men in Brazil: a respondent-driven sampling survey | Brazil | - To estimate the prevalence of sexual violence experienced by MSM in Brazil | - Qualitative  - Survey | N = 3,859  LGBQ+ men |
| Salim, McConnell & Messman-Moore (2020) | Bisexual women’s experiences of stigma and verbal sexual coercion: The roles of internalized heterosexism and outness | United States | - To investigate the relation between bisexual-specific stigma and verbal sexual coercion among bisexual women. | - Quantitative  - Survey | N = 350  LGBQ+ women |
| Satinsky & Jozkowski Kristen (2014) | Sexual coercion and behavior among a sample of sexual minority women | United States | - To examine rates of sexual coercion among sexual-minority women and compare this to their health status and happiness level. | - Quantitative  - Survey | LGBQ+ women; Transgender people |
| Schuyler, Klemmer, Mamey, Schrager, Goldbach, Holloway & Castro (2020) | Experiences of sexual harassment, stalking, and sexual assault during military service among LGBT and non-LGBT service members | United States | - To examine experiences of sexual harassment, stalking, and sexual assault victimization during service in a sample of LGBT and non-LGBT active-duty service members | - Quantitative  - Survey | N = 544  LGBQ+ women; LGBQ+ men; Transgender people; Heterosexual women; Heterosexual men |
| Seabrook, McMahon, Duquaine, Johnson, DeSilva (2018) | Sexual assault victimization and perceptions of university climate among bisexual women | United States | - To examine victimization rates, sense of community, and perceived university and student responsiveness to sexual violence among bisexual women compared to heterosexual and GL women. | - Quantitative  - Survey | N = 6,067  LGBQ+ women; Heterosexual women |
| Seelman, Kristie L. (2015) | Unequal treatment of transgender individuals in domestic violence and rape crisis programs | United States | - To examine whether transgender people are more likely to face discrimination by domestic violence and rape crises services on the basis of economic and demographic factors. | - Quantitative  - Survey | N = 2,424  Transgender people |
| Semple, Stockman, Goodman-Meza, Pitpitan, Strathdee, Chavarin, Rangel, Torres, Patterson (2017) | Correlates of sexual violence among men who have sex with men in Tijuana, Mexico | Mexico | - To examine correlates of sexual violence victimization amongst MSM in Tijuana, Mexico | - Quantitative  - Survey | N = 201  LGBQ+ men |
| Shaw, Lorway, Deering, Avery, Mohan, Bhattacharjee, Reza-Paul, Isac, Ramesh, Washington, Moses & Blanchard (2012) | Factors associated with sexual violence against men who have sex with men and transgendered individuals in Karnataka, India | Other: India | - To examine the rates of sexual violence among MSM and transgender individuals in Southern India and the association with health related behaviours and sexual practices. | - Quantitative  - Survey | N = 543  LGBQ+ men; Transgender people |
| Sigurvinsdottir & Ullman (2016b) | Sexual orientation, race, and trauma as predictors of sexual assault recovery | United States | - To examine the recovery trajectories of sexually assaulted women and what role sexual orientation, race, and revictimization play in that recovery. | - Quantitative  - Longitudinal surveys | N = 905  LGBQ+ women; Heterosexual women |
| Sigurvinsdottir & Ullman (2016a) | Sexual assault in bisexual and heterosexual women survivors | United States | - To examine the relationship between social support and psychological outcomes following sexual assault, and how that relationship is impacted by sexual orientation. | - Quantitative  - Longitudinal surveys | N = 905  LGBQ+ women; Heterosexual women |
| Sigurvinsdottir & Ullman (2015) | The role of sexual orientation in the victimization and recovery of sexual assault survivors | United States | - To examine the impact of sexual assault on lesbian and bisexual women. | - Quantitative  - Survey | N = 1,863  LGBQ+ women; Heterosexual women |
| Smidt, Rosenthal, Smith & Freyd (2021) | Out an in harm's way: Sexual minority students' psychological and physical health after institutional betrayal and sexual assault | United States | - To examine whether sexual minority individuals experienced difference rates of sexual violence victimization compared to heterosexual students. | - Quantitative  - Survey | N = 899  LGBQ+ women; LGBQ+ men |
| Snyder, Scherer & Fisher (2018) | Interpersonal violence among college students: Does sexual orientation impact risk of victimization? | United States | - To examine the prevalence of interpersonal violence across sexual orientations and examine whether sexual orientation was a predictor of interpersonal violence. | - Quantitative  - Survey | N = 43,000  LGBQ+ women; LGBQ+ men; Heterosexual women; Heterosexual men |
| Solomon, Combs, Allen, Roles, DiCarlo, Reed & Klaver (2021) | The impact of minority stress and gender identity on PTSD outcomes in sexual minority survivors of interpersonal trauma | Online | - To examine how the minority stress model can be applied to a number of mental health concerns and specific populations and the development of PTSD following experiences of interpersonal trauma. | - Quantitative  - Survey | N = 197  LGBQ+ women; LGBQ+ men; Transgender people |
| Staples & Fuller (2021) | Adult sexual assault severity among transgender people of color: The impact of double marginalization | United States | - To investigate the effect of gender expression and race on adult sexual assault severity. | - Quantitative  - Survey | N = 2345  Transgender people |
| Stoddard, Dibble & Fineman (2009) | Sexual and physical abuse: A comparison between lesbians and their heterosexual sisters | United States | - To compare experiences of abuse (CSA and ASA) between lesbians and their heterosexual sisters | - Quantitative  - Survey | N = 648  LGBQ+ women; Heterosexual women |
| Strike, Myers, Calzavara & Haubrich (2001) | Sexual coercion among young street-involved adults: Perpetrators' and victims' perspectives | Canada | - To explore sexual coercion in dating situations among young adults aged 18-25 | - Qualitative  - Focus groups | N = 50  LGBQ+ women; LGBQ+ men; Heterosexual Women; Heterosexual Men |
| Tilapaugh (2016) | Resisting erasure: Critical influences for men who survived sexual violence in higher education | United States | - To explore the experiences of cisgender and transgender men who experienced sexual violence in US colleges and universities | - Qualitative  - Interviews | LGBQ+ men; Transgender men; Heterosexual Men |
| Tilley, Kolodetsky, Cottrell & Tilton (2020) | Correlates to Increased Risk of Sexual Assault and Sexual Harassment Among LGBT+ University Students | United States | - To examine the correlates to sexual assault and other forms of sexual misconduct of LGBT+ college students. | - Quantitative  - Survey | N = 6,973  LGBQ+ women; LGBQ+ men; Transgender people; Heterosexual women; Heterosexual men |
| Toro-Alfonso & Rodríguez-Madera (2004) | Sexual coercion in a sample of Puerto Rican gay males | Puerto Rico | - To examine the experiences of Puerto Rican gay men with domestic violence and sexual coercion in the context of the HIV epidemic. | - Quantitative  - Survey | N = 302  LGBQ+ men |
| Twinley (2017) | Woman-to-woman rape and sexual assault, and its impact upon the occupation of work: Victim/survivors life roles of worker or student as disruptive and preservative | UK | - The study explored the perceived impacts of woman-to-woman rape and sexual assault and the aftermath for victim/survivors experience of their occupation. | - Mixed  - Survey  - Interviews | N = 159  LGBQ+ women; Heterosexual women |
| Ussher, Hawkey, Perz, Liamputtong, Sekar, Marjadi, Schmied, Dune & Brook (2020) | Crossing boundaries and fetishization: Experiences of sexual violence for trans women of color | Australia | - To examine subjective experiences of sexual violence for trans women of color living in Australia. | - Qualitative  - Interviews | N = 31  Transgender women |
| VanderLaan & Vasey (2009) | Patterns of sexual coercion in heterosexual and non-heterosexual men and women | Canada | - To compare the sexually coercive behavior of heterosexual and non-heterosexual men and women | Quantitative | N = 414  LGBQ+ women; LGBQ+ men; Heterosexual women; Heterosexual men |
| Waldner-Haugrud & Gratch (1997) | Sexual coercion in gay/lesbian relationships: Descriptives and gender differences | United States | - To measure the frequency of sexually coercing acts in gay and lesbian relationships | - Quantitative  - Survey | N = 273  LGBQ+ women; LGBQ+ men |
| Watson, Craney, Greenwalt, Beaumont, Whitney & Flores (2021) | "I was a game or a fetish object": Diverse bisexual women's sexual assault experiences and effects on bisexual identity | United States | - To explore sexual assault experiences among diverse bisexual women and gender expansive people. | - Mixed  - Survey  - Qualitative questions | LGBQ+ women; Gender expansive people |
| Wells, Starks, Robel, Kelly, Parsons & Golub (2016) | From sexual assault to sexual risk: A relational pathway? | United States | - To evaluate the role of perceived partner pressure as a mediator between ASA history and sexual risk behavior among women and gay/bisexual men | - Quantitative  - Survey | N = 3014  LGBQ+ women; LGBQ+ men; Heterosexual women |
| Wilkerson, Di Paola, Nieto, Schick, Latini, Braun-Harvey, Zoschke, McCurdy (2021) | Sexual violence and chemsex among substance-using sexual and gender minorities in Texas | United States | - To understand how sexual assault co-occurs with drug-use and whether chemsex plays a role in this | - Mixed  - Survey  - Interviews | N = 1,273  LGBQ+ women; LGBQ+ men; Transgender people |
| Wilson, Simpson, Butler, Yap, Richters & Donovan (2017) | “You’re a woman, a convenience, a cat, a poof, a thing, an idiot: Transgender women negotiating sexual experiences in men’s prisons in Australia | Australia | - To examine the lived experience of transgender women in Australian men's and women's prisons. | - Qualitative  - Interviews | N = 7  Transgender women |
